# In Alzheimer-prone brain regions, metabolism and risk-gene expression are strongly correlated

**DOI:** 10.1093/braincomms/fcac216

**Published:** 2022-08-25

**Authors:** Fengdan Ye, Quentin Funk, Elijah Rockers, Joshua M Shulman, Joseph C Masdeu, Belen Pascual, for the Alzheimer’s Disease Neuroimaging Initiative

**Affiliations:** Department of Physics and Astronomy, Rice University, Houston, TX 77005, USA; Center for Theoretical Biological Physics, Rice University, Houston, TX 77005, USA; Nantz National Alzheimer Center, Houston Methodist Neurological and Research Institute, Houston Methodist Hospital, Weill Cornell Medicine, Houston, TX 77030, USA; Nantz National Alzheimer Center, Houston Methodist Neurological and Research Institute, Houston Methodist Hospital, Weill Cornell Medicine, Houston, TX 77030, USA; Nantz National Alzheimer Center, Houston Methodist Neurological and Research Institute, Houston Methodist Hospital, Weill Cornell Medicine, Houston, TX 77030, USA; Department of Neurology, Baylor College of Medicine, Houston, TX 77030, USA; Department of Neuroscience, Baylor College of Medicine, Houston, TX 77030, USA; Department of Molecular and Human Genetics, Baylor College of Medicine, Houston, TX 77030, USA; Center for Alzheimer’s and Neurodegenerative Diseases, Baylor College of Medicine, Houston, TX 77030, USA; Jan and Dan Duncan Neurological Research Institute, Texas Children’s Hospital, Houston, TX 77030, USA; Nantz National Alzheimer Center, Houston Methodist Neurological and Research Institute, Houston Methodist Hospital, Weill Cornell Medicine, Houston, TX 77030, USA; Nantz National Alzheimer Center, Houston Methodist Neurological and Research Institute, Houston Methodist Hospital, Weill Cornell Medicine, Houston, TX 77030, USA

**Keywords:** AHBA gene expression, brain metabolism, Alzheimer’s disease, *APOE*, *SORL1*

## Abstract

Neuroimaging in the preclinical phase of Alzheimer’s disease provides information crucial to early intervention, particularly in people with a high genetic risk. Metabolic network modularity, recently applied to the study of dementia, is increased in Alzheimer’s disease patients compared with controls, but network modularity in cognitively unimpaired elderly with various risks of developing Alzheimer’s disease needs to be determined. Based on their 5-year cognitive progression, we stratified 117 cognitively normal participants (78.3 ± 4.0 years of age, 52 women) into three age-matched groups, each with a different level of risk for Alzheimer’s disease. From their fluorodeoxyglucose PET we constructed metabolic networks, evaluated their modular structures using the Louvain algorithm, and compared them between risk groups. As the risk for Alzheimer’s disease increased, the metabolic connections among brain regions weakened and became more modular, indicating network fragmentation and functional impairment of the brain. We then set out to determine the correlation between regional brain metabolism, particularly in the modules derived from the previous analysis, and the regional expression of Alzheimer-risk genes in the brain, obtained from the Allen Human Brain Atlas. In all risk groups of this elderly population, the regional brain expression of most Alzheimer-risk genes showed a strong correlation with brain metabolism, particularly in the module that corresponded to regions of the brain that are affected earliest and most severely in Alzheimer’s disease. Among the genes, *APOE* and *CD33* showed the strongest negative correlation and *SORL1* showed the strongest positive correlation with brain metabolism. The Pearson correlation coefficients remained significant when contrasted against a null-hypothesis distribution of correlation coefficients across the whole transcriptome of 20 736 genes (*SORL1*: *P* = 0.0130; *CD33*, *P* = 0.0136; *APOE*: *P* = 0.0093). The strong regional correlation between Alzheimer-related gene expression in the brain and brain metabolism in older adults highlights the role of brain metabolism in the genesis of dementia.

## Teaser

In Alzheimer-disease-prone brain regions of elderly at risk and Alzheimer’s patients, Ye *et al*. found a strong correlation between Alzheimer-related gene expression and brain metabolism. Additionally, weakened metabolic connections between brain regions were observed for high-risk individuals. The findings highlight the importance of brain metabolism in the genesis of dementia.

## Graphical Abstract

**Graphical Abstract:**
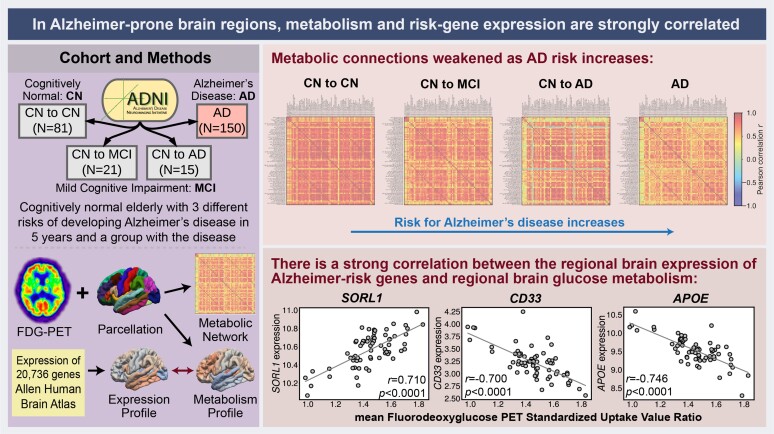

## Introduction

Alzheimer’s disease affects regional brain glucose metabolism early in the course of the disease and with a characteristic anatomic distribution, including posterior cingulate and temporo-parietal cortex.^1,2^ Measured *in vivo* with fluorodeoxyglucose (^18^F-FDG) PET, regional metabolism has been used to predict the conversion of mild cognitive impairment (MCI) to Alzheimer’s disease and even the likelihood that a cognitively normal (CN) individual will develop MCI or, later, Alzheimer’s disease.^3,4^

Construction and analysis of brain metabolic networks using FDG PET have recently gained momentum in studying MCI and Alzheimer’s disease.^5–7^ Here, we used this technique to study CN individuals separated in groups with different risk of progression. We aimed to determine whether the strength of the brain metabolic connections could clarify aetiology and predict prognosis. This analysis yielded several metabolic brain modules, one of which included the areas of the brain where neuronal loss begins and is most extensive in Alzheimer’s disease, such as the medial temporal lobe.

Next, we set to define whether metabolism in these modules is genetically determined. More than 25 risk genes have been identified for Alzheimer’s disease, with *APOE4* contributing the highest risk.^8^ Besides examining the association of genetic variants with risk for Alzheimer’s disease,^9^ several post-mortem studies of human brain report differential expression of implicated candidate genes in Alzheimer’s disease, including *APOE* and *CD33.*^10,11^ Many studies have explored brain imaging findings, including brain metabolism, in cognitively unimpaired individuals with various genotypes related to risk for Alzheimer’s disease, particularly of *APOE*.^12–17^ However, there is a dearth of studies focusing on the relationship between the anatomic distribution in the brain of risk-gene expression and molecules related to Alzheimer’s disease, such as β amyloid and tau,^18,19^ and none related to brain metabolism, which is likely key in the pathogenesis of Alzheimer’s disease.^20^ To determine whether risk-gene expression in various regions of the brain, and, particularly in the modules identified above, has an effect on brain metabolism, we used the Allen Human Brain Atlas (AHBA)^21^ to compare the regional gene expression of the main Alzheimer-risk genes with regional brain metabolism determined by ^18^F-FDG PET.

## Material and methods

### Neuroimaging datasets and pre-processing

The present study first analysed metabolic data from cognitively unimpaired individuals in the Alzheimer’s Disease Neuroimaging Initiative (ADNI) database (adni.loni.usc.edu). The ADNI was launched in 2003 as a public–private partnership, led by Principal Investigator Michael W. Weiner, MD. The primary goal of ADNI has been to test whether serial MRI, PET, other biological markers and clinical and neuropsychological assessment can be combined to measure the progression of MCI and early Alzheimer’s disease. For up-to-date information, see www.adni-info.org.

FDG PET scans collected when the participants had a CN diagnosis were selected. Depending on the diagnosis in the subsequent 5 years, scans were filtered into three groups: participant remained CN (‘CN to CN’), participant progressed to MCI but not Alzheimer’s disease (‘CN to MCI’), and participant progressed to Alzheimer’s disease (‘CN to AD’). Participants that lacked sufficient data to determine the 5-year progression were discarded. In addition, we studied an Alzheimer’s disease patient group (‘AD’) consisting of participants that were already diagnosed with Alzheimer’s disease at baseline and selected the FDG PET scans closest to the participants’ baseline evaluation dates. For the ‘AD’ group, only participants with *APOE* genotype 3/3, 3/4, or 4/4 were considered. A diagram illustrating the pipeline of data collection, classification and inspection is provided in the Supplementary Fig. 1.

All FDG PET scans were downloaded from ida.loni.usc.edu in their fully pre-processed form. These pre-processed FDG PET scans had already gone through frame alignment and averaging and had been reoriented into a standard 160 × 160 × 96 voxel image grid, with 1.5 mm cubic voxels. All images were also smoothed to a uniform isotropic resolution of 8 mm full width at half maximum, the approximate resolution of the lowest resolution scanners used in ADNI. Using the pre-processed FDG PET scans vastly reduced the heterogeneity in the ADNI data, which were obtained using different scanners and reconstruction protocols.

The closest-in-time T_1_-weighted MRI to each FDG PET scan was downloaded in the original format and was processed using FreeSurfer 5.3 to obtain cortical parcellation and subcortical segmentation. The quality of the parcellation and segmentation was carefully inspected. If the quality proved unsatisfactory, other repeats of the same MRI scan session were inspected. If no repeat exhibited acceptable quality, an alternative FDG PET scan from the same participant was chosen if it belonged to the same group, and its closest-in-time T_1_-weighted MRI was inspected as described above. If all alternatives failed, the participant was removed from the dataset.

Each FDG PET scan was co-registered to its corresponding T_1_-weighted MRI using SPM12 in MATLAB R2019b. The goodness of alignment between the co-registered FDG PET and T_1_ MRI was then visually assessed, and the quality of the FDG PET scans was examined to make sure no anomaly existed. Any images that failed the inspection were discarded. The T_1_-weighted MRI was then normalized to the standard space using SPM12, bringing along its parcellation/segmentation and the co-registered FDG PET. The participant-specific pons mask was obtained by finding the overlap between the participant-specific brainstem and a general pons mask in standard space. The quality of the normalization and the accuracy of the pons mask were then visually inspected. The average standardized uptake values (SUV) in the pons were then obtained for each FDG PET scan, and SUV ratio (SUVR) levels were obtained by dividing the SUV of each and all brain voxels by the pons average.

Given the large size of the ADNI database and its multi-site nature, it is essential to verify the data quality and the diagnosis of the participants. As mentioned above, FY inspected the quality of T_1_-weighted MRI and FDG PET scans, as well as the segmentation output by FreeSurfer. Two clinicians (BP and JM) carefully reviewed the diagnosis of each participant in the ‘CN to MCI’, ‘CN to AD’ and ‘AD’ groups. Data reviewed for each participant included age, clinical dementia rating sum of boxes (CDR-SB), and its components, Alzheimer’s disease assessment Scale 11 tasks (ADAS11), 13 tasks (ADAS13), mini-mental state exam (MMSE), Montreal Cognitive Assessment (MOCA), average FDG PET SUVR (of angular, temporal and posterior cingulate cortex), amyloid positivity, CSF Aβ, CSF Tau, CSF p-Tau, NPI-A (delusions), NPI-B (hallucinations), as well as all FDG PET and T_1_-weighted MRI scans available from each potential participant in our study throughout their participation in ADNI. Any participant that did not show signs of MCI or Alzheimer’s disease in the 5-year progression or showed signs of other types of dementia (e.g. Lewy Body Dementia) was removed from the ‘CN to MCI’ or ‘CN to AD’ group. Similarly, any participant that did not show any patterns for Alzheimer’s disease or showed patterns for other types of dementia were removed from the ‘AD’ group. Participant removal was a consensus process, with agreement by both clinicians. The ‘CN to CN’ group was not reviewed to this extent as its definition was the most straightforward and controlled, leaving less room for error.

After all inspections were finished, the ‘CN to CN’ and ‘CN to MCI’ groups were age-matched to the ‘CN to AD’ group, removing the youngest participants until the mean ages matched. No age-matching was done on the ‘AD’ group. In the end, there were 81 participants in the ‘CN to CN’ group, 21 participants in the ‘CN to MCI’ group, 15 participants in the ‘CN to AD’ group, and 150 participants in the ‘AD’ group. Age, sex, years of education, MMSE, MOCA, CDR-SB, ADAS11, ADAS13 and amyloid positivity were calculated for each group and significance testing was carried out for demographics. Note that age was calculated at the time the FDG PET scan was collected. Amyloid positivity was calculated from Florbetapir or Pittsburgh compound B (PIB) PET data available within 1 year from the FDG PET scan date (and 5 years later). ADNI provided Florbetapir and PIB scores, i.e. average SUVR in regions of interest (ROI), and the corresponding thresholds to determine positivity. A participant was considered amyloid positive if any Florbetapir and/or PIB PET scans within 1 year scored above the pre-determined threshold (Florbetapir threshold: 1.11 if normalized by whole cerebellum, 0.79 if normalized by composite reference region; PIB threshold: 1.50). Participants who did not have Florbetapir or PIB PET data available within 1 year from FDG PET scan date (and 5 years later) were excluded from the calculation of positive amyloid PET ratio. MMSE, MOCA, CDR-SB, ADAS11 and ADAS13 were calculated from data available within 90 days from FDG PET imaging date (and 5 years later). Participants who did not have MMSE, MOCA, CDR-SB, ADAS11 and ADAS13 evaluation within 90 days from FDG PET imaging date (and 5 years later) were excluded from the corresponding calculation of mean and standard deviation.

### Construction and clustering of whole-brain FDG PET network

A whole-brain FDG PET network was constructed for each group with 72 ROIs as nodes, which included the 68 cortical regions in the Desikan-Killiany cortical atlas,^22^ plus hippocampus and amygdala bilaterally, as segmented by Freesurfer. A full list of the 72 ROIs can be found in Supplementary Tables 3–15. The mean SUVR was obtained for each ROI and the FDG PET network was then constructed by calculating the Pearson correlation between all pairs of ROIs across all participants in the group. The method produced a 72 by 72 correlation matrix for each group. Sex-specific correlation matrices were also obtained.

The Louvain algorithm^23^ was subsequently run on each correlation matrix to cluster the FDG PET networks. The algorithm partitions ROIs into modules to maximize modularity, *M*. This maximization process clusters regions with strong connections into the same module, and regions with weak connections into separate modules. Since the correlation matrix can contain negative correlations, we adopted a modified definition of modularity specifically designed for correlation matrices derived from neuroimaging data^24^:

$$\begin{array}{rl} M\;= & M^{+}+\frac{v^{-}}{v^{+}+v^{-}}M^{-}=\frac{1}{v^{+}}\underset{1\leq i,j\leq n}{\sum }\;\left(w_{ij}^{+}-\frac{s_{i}^{+}s_{j}^{+}}{v^{+}}\right)\delta (\sigma_{i},\sigma_{j})\;\\ & -\frac{1}{v^{+}+v^{-}}\underset{1\leq i,j\leq n}{\sum }\;\left(w_{ij}^{-}-\frac{s_{i}^{-}s_{j}^{-}}{v^{-}}\right)\delta (\sigma_{i},\sigma_{j}), \end{array}\;$$

where

$$M^{+}=\frac{1}{v^{+}}\underset{1\leq i,j\leq n}{\sum }\;\left(w_{ij}^{+}-\frac{s_{i}^{+}s_{j}^{+}}{v^{+}}\right)\delta (\sigma_{i},\sigma_{j})$$

and

$$M^{-}=-\frac{1}{v^{-}}\underset{1\leq i,j\leq n}{\sum }\;\left(w_{ij}^{-}-\frac{s_{i}^{-}s_{j}^{-}}{v^{-}}\right)\delta (\sigma_{i},\sigma_{j}).$$

The weight of a positive connection between nodes *i* and *j* is denoted as $w_{ij}^{+}\in (0,1),\;w_{ij}^{-}\;=\;0$. In the present study, $w_{ij}^{+}$ is the value of the Pearson correlation coefficient. The weight of a negative connection between nodes *i* and *j* is denoted as $w_{ij}^{-}\in (0,1),\;w_{ij}^{+}=0$, where $w_{ij}^{-}$ is the absolute value of the Pearson correlation coefficient. The strength of node *i* is the sum of positive or negative weights of *i*, $s_{i}^{\pm }=\underset{j}{\sum }\;w_{ij}^{\pm }$. The total weight of the network is the sum of all positive or negative connection weights, $v^{\pm }=\underset{ij}{\sum }\;w_{ij}^{\pm }$. Due to matrix symmetry, connection weights are counted twice for each connection when calculating *v*^±^. Total number of nodes *n* = 72. *δ*(*σ*_*i*_, *σ*_*j*_) = 1 if node *i* and *j* are in the same module, and *δ*(*σ*_*i*_, *σ*_*j*_) = 0 otherwise.

This definition of modularity supports placement of positively connected pairs of nodes in the same module, and placement of negatively connected pairs of nodes in distinct modules. Maximization of *M* is a balance between maximizing *M*^+^, which encourages placement of positive connections within modules, and maximizing *M*^−^, which encourages placement of negative connections between modules. The contribution of *M*^−^ to this balance is proportional to the ratio of negative links [(*v*^−^)/(*v*^+^ + *v*^−^)]. If there is zero negative connection in the network, the maximization of *M* is entirely dependent on maximization of *M*^+^. If there are equal number of negative and positive connections, then *M*^+^ has twice the influence as *M*^−^. Therefore, this modularity definition assumes that negative connections only play an auxiliary role in network structure, as compared with positive connections.^24^

The Louvain algorithm is a stochastic process. Running the Louvain algorithm multiple times might lead to slightly different partitions of the same network. To find the stable partition that multiple runs of Louvain algorithm converge to, we adopted the method of consensus clustering.^25^ The Louvain algorithm was run 100 times for the same network, and subsequently 100 partitions were obtained. A 72 by 72 agreement matrix *A* was then established, where *A*_*ij*_ was the frequency where node *i* and node *j* were assigned the same module across the 100 partitions. Note that the Louvain algorithm outputs a set of hierarchical partitions and the lowest hierarchical level partition was used to generate the agreement matrix. This is because the lowest hierarchical level of the Louvain output has been shown to have better performance than higher hierarchical outputs on a set of benchmark networks.^26,27^ The Louvain algorithm was then run on the agreement matrix. This produced another set of 100 partitions from which a new agreement matrix was built. The above process was repeated with the new agreement matrix until the partitions had converged to a single stable partition. We report the stable partition and the corresponding modularity value.

Implementation of the method described above was provided by the Brain Connectivity Toolbox (http://www.brain-connectivity-toolbox.net)^28^ and was run in MATLAB R2019b.

### Gene expression dataset

To determine gene expression in the modules calculated above, we used the AHBA.^21^ The AHBA provides regional transcription profiles of 20 736 protein-coding genes, based on a complete transcriptome dataset consisting of 58 692 measurements of gene expression in 3702 brain samples obtained from six individuals. French and Paus^29^ converted the raw AHBA data to a median expression profile across donors in the Desikan-Killiany atlas. The present study used this converted AHBA gene expression data for analysis.

We studied genes that have been previously reported to be related to Alzheimer’s disease^30–32^ and especially those in Sepulcre *et al*.^18^ We further filtered the genes by only keeping the ones with high consistency score (>0.446) in AHBA, as described in French and Paus.^29^ A gene expression profile with higher consistency score is more representative of the six donors. Ten Alzheimer-risk genes remained after filtering: *APOE*, *BIN1*, *CD33*, *CLU*, *CELF1* (previously known as *CUGBP1*), *MAPT*, *MEF2C*, *FERMT2* (previously known as *PLEKHC1*), *SORL1* and *TREM2*.

A mean FDG profile was obtained for each group by averaging the mean FDG PET SUVR for each ROI across all participants in the group. Pearson correlation was then calculated between the median gene expression profile and the mean FDG PET SUVR across ROIs.

### Statistical analysis

Two-sample two-tailed *t*-tests with unequal variance were used to determine significant age differences between men and women in each group and between groups, as well as significant differences in years of education between groups. The two-tailed *z*-test for proportions was used to determine significant sex differences between groups.

Two-tailed *P*-values for the Pearson correlation between gene expression and mean FDG PET SUVR are reported. To better gauge the significance of the correlations, Pearson *r*-values were contrasted against a null-hypothesis distribution of correlation coefficients across the whole transcriptome of 20 736 genes. An effective one-tailed *P*-value *p_e_* was calculated for each correlation from its corresponding *z*-value in the null distribution.

### Data availability

The data that support the findings of this study are openly available in the ADNI database at adni.loni.usc.edu. The list of participant IDs used in the analysis as well as all code that was developed by the authors will be available upon direct request to the corresponding author and review by all authors.

## Results

### Key descriptive statistics for participant groups

**Figure 1:**
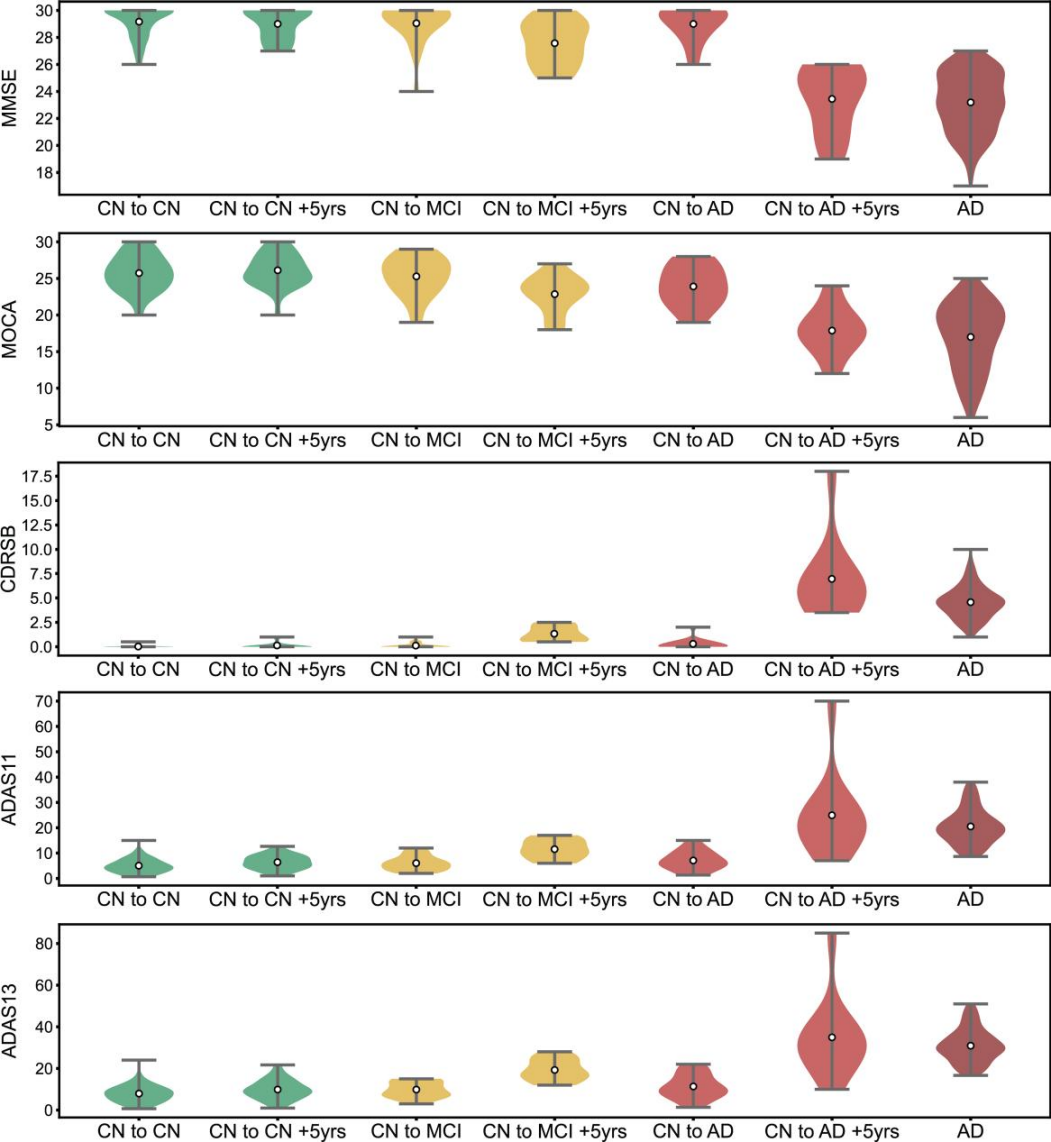
**Cognitive tests MMSE, MOCA, CDR-SB, ADAS11 and ADAS13 scores at baseline and 5 years later.** The size of each group is listed in Table 1. For each test, the *y*-axis represents the participants’ raw scores in the respective test. The grey whiskers show extrema, while the white circles represent mean. Coloured areas show the distribution of values. ADAS11, Alzheimer’s disease assessment Scale 11 tasks; ADAS13, Alzheimer’s disease assessment Scale 13 tasks; CDR-SB, clinical dementia rating sum of boxes; MMSE, mini-mental state examination; MOCA, Montreal Cognitive Assessment.

Age, sex, years of education, MMSE, MOCA, CDR-SB, ADAS11, ADAS13 and amyloid positivity for each group are summarized in Table 1. Violin plots of MMSE, MOCA, CDR-SB, ADAS11 and ADAS13 scores at baseline and 5 years later are shown in Fig. 1. No significant differences in demographics were found between the three CN groups. However, there were significant differences between the Alzheimer’s disease group and the CN groups (Supplementary Table 1).

**Table 1:** Key descriptive statistics for each group

		CN to CN		CN to MCI		CN to AD		AD
*Demographics*								
Number of participants		81		21		15		150
Sex (women/men)		35/46		9/12		8/7		72/78
Age of women		78.2 (3.7)		78.4 (3.2)		79.0 (4.6)		71.5 (7.7)
Age of men		78.4 (4.5)		78.1 (3.7)		77.5 (4.8)		74.7 (7.6)
Age (women and men)		78.3 (4.1)		78.2 (3.4)		78.3 (4.6)		73.2 (7.8)
Years of education		16.5 (2.6)		15.8 (2.0)		16.4 (2.9)		15.4 (2.5)
*Amyloid positivity and neuropsychological tests*								
		**Baseline**	**+5 years**	**Baseline**	**+5 years**	**Baseline**	**+5 years**	**Baseline**
Positive amyloid PET	Ratio	32.2%	53.4%	88.2%	100%	91.7%	100%	100%
	Participants available	59	58	17	14	12	7	91
MMSE	Mean (s.d.)	29.165 (1.152)	29.000 (1.144)	29.048 (1.463)	27.571 (1.591)	29.000 (1.211)	23.444 (2.362)	23.189 (2.216)
	Participants available	79	26	21	14	15	9	148
MOCA	Mean (s.d.)	25.737 (2.432)	26.120 (2.233)	25.286 (2.630)	22.857 (2.503)	23.909 (2.678)	17.875 (3.219)	17.000 (4.531)
	Participants available	57	25	14	14	11	8	94
CDR-SB	Mean (s.d.)	0.025 (0.110)	0.135 (0.327)	0.119 (0.263)	1.333 (0.767)	0.300 (0.510)	6.950 (3.984)	4.561 (1.660)
	Participants available	79	26	21	15	15	10	148
ADAS11	Mean (s.d.)	5.034 (2.794)	6.410 (2.794)	6.031 (2.804)	11.548 (3.673)	7.089 (3.669)	24.933 (15.960)	20.515 (6.856)
	Participants available	79	26	21	14	15	10	147
ADAS13	Mean (s.d.)	7.933 (4.161)	9.872 (4.620)	9.841 (3.694)	19.262 (5.034)	11.355 (5.716)	34.933 (18.350)	30.913 (8.022)
	Participants available	79	26	21	14	15	10	145

In parenthesis are standard deviations. +5 years: data 5 years after baseline. Please see Section 2.1 for the timing and determination of age, amyloid positivity and cognitive data. No significant differences in demographics were found between the three CN groups (Supplementary Table 1). No significant differences in age between men and women were found within the three CN groups. For ‘AD’ group, men were significantly older than women.

### Whole-brain FDG PET network and its structure

**Figure 2:**
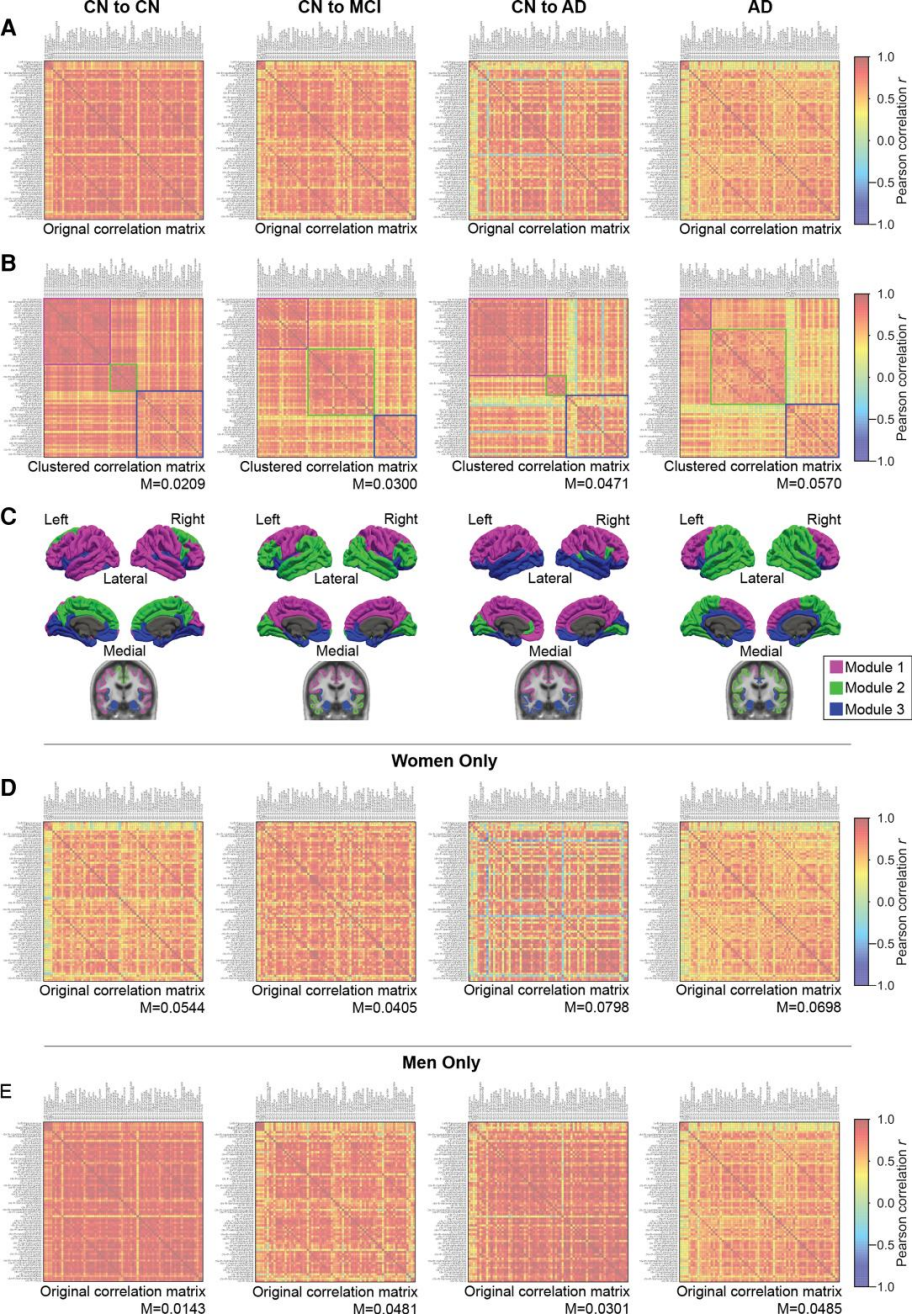
**FDG PET network and modular structure for each group.** (**A**) Original correlation matrices. Pearson correlation coefficients were calculated across subjects from mean FDG PET SUVR between 72 ROIs. The 72 ROIs start with left-hippocampus, left-amygdala, right-hippocampus and right-amygdala, followed by 34 Desikan-Killiany cortical regions on the left hemisphere (‘lh-’) and then 34 Desikan-Killiany cortical regions on the right hemisphere (‘rh-’). A left-hemisphere region usually had high correlation with its right-hemisphere counterpart, resulting in two prominent off-diagonal lines in each of the matrices. (**B**) The re-arranged correlation matrices to reflect the clustered structure of the FDG PET networks. ROIs are ordered by module allegiance. The coloured squares represent modules. (**C**) Brain maps of modules. The colours here match the coloured squares in B. (**D**) The original correlation matrices, from women only. Modularity for the optimal partition is provided in text right below the matrices. (**E**) The original correlation matrices, from men only. Modularity for the optimal partition is provided in text right below the matrices. A full list of the 72 ROIs in the matrices can be found in Supplementary Tables 3–15.

Most connections in the whole-brain FDG PET correlation matrices for the four groups (Fig. 2A) were positive. The strongest negative correlation was only –0.453, as compared with the strongest positive correlation at 0.990.

The overall connection strength weakened as risk for Alzheimer’s disease increased. Notably, in the ‘CN to CN’ group, the entorhinal cortex of both hemispheres had the weakest connection with the rest of the network. The same observation held true for the ‘CN to AD’ group, except the correlation was now negative. Overall, hippocampus and amygdala had weaker connections with the neocortical brain as compared with the connections between cortical regions (see the yellow ‘stripes’ in the first four rows and columns in all four matrices, Fig. 2A). As risk for Alzheimer’s disease increased, however, some connections within cortical regions became as weak as the hippocampus-cortex and amygdala-cortex connections. In the Alzheimer’s disease patient group, the entire network was weak and only a few connections remained strong.

The Louvain algorithm yielded three modules in all four groups (Fig. 2B), which were mapped onto 3D brain (Fig. 2C). The module composition differed across groups, however, the regions first affected by Alzheimer’s disease, such as hippocampus, amygdala, and entorhinal cortex were always included in Module 3. As inter-module connections weakened, the whole-brain FDG PET network became monotonically more modular from the ‘CN to CN’ group (*M* = 0.0209) to the ‘AD’ group (*M* = 0.0570).

Sex impacted the modular organization of the networks (Fig. 2D–E). In the ‘CN to CN’, ‘CN to AD’ and ‘AD’ groups, the metabolic networks of women were weaker and more modular, that is, more fragmented, than those of men. The ‘CN to MCI’ group did not show significant difference between women and men. The clustered correlation matrices and the mapping of modules onto brain for both sexes are provided in Supplementary Fig. 2. Two-tailed *t*-test found no significant differences in age between men and women within each group, except for the ‘AD’ group where men were significantly older than women (men: 74.7 ± 7.6, women: 71.5 ± 7.7, *P*-value = 0.0097). ‘CN to CN’ (men: 78.4 ± 4.5, women: 78.2 ± 3.7, *P*-value = 0.85), ‘CN to MCI’ (men: 78.1 ± 3.7, women: 78.4 ± 3.2, *P*-value = 0.80) and ‘CN to AD’ (men: 77.5 ± 4.8, women: 79.0 ± 4.6, *P*-value = 0.55) did not reach significance in *t*-tests.

### Correlation between brain gene expression and metabolism

**Figure 3:**
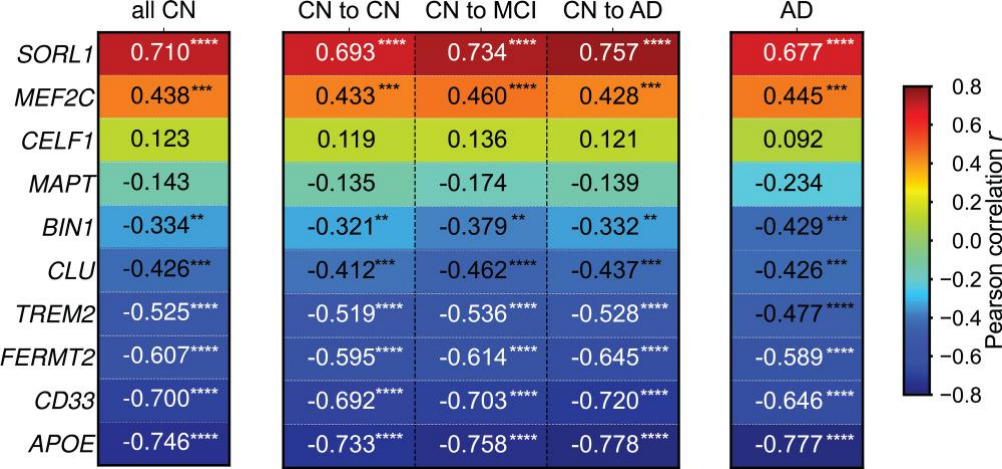
**Pearson correlation between the mRNA expression of Alzheimer-risk genes and brain metabolism.** Brain metabolism was calculated as the mean FDG PET SUVR averaged across all participants in each group. Genes were ordered based on correlation strength, from most positive to most negative. Significance level is defined as: *P ≤ 0.05, **P ≤ 0.01, ***P ≤ 0.001, ****P ≤ 0.0001.

The mRNA expression of most Alzheimer-related genes correlated strongly with the mean FDG PET SUVR in all four groups, as well as a combination of all CN groups (‘CN to CN’, ‘CN to MCI’ and ‘CN to AD’ combined, referred to as ‘all CN’ group; Fig. 3). In all groups, *APOE* showed the strongest negative correlation with FDG PET SUVR (*r* ≤ −0.733, *P* < 0.0001), and *SORL1* showed the strongest positive correlation with FDG PET SUVR (*r* ≥ 0.677, *P* < 0.0001). Similar analyses were carried out for participants by *APOE* genotype but no significant correlations were found (Supplementary Fig. 3).

All groups followed the same correlation pattern: (i) all genes showed significant correlations with FDG PET SUVR, except for *CELF1* and *MAPT*; (ii) *SORL1* and *MEF2C* positively correlated with FDG PET SUVR, while (iii) *BIN1*, *CLU*, *TREM2*, *PLEKCH1*, *CD33*, and *APOE* negatively correlated with FDG PET SUVR. The absolute values of the correlation between *APOE* expression and FDG PET SUVR monotonically increased from ‘CN to CN’ group (−0.733) to ‘CN to MCI’ group (−0.758) and to ‘CN to AD’ group (−0.778) (Fig. 3).

**Figure 4:**
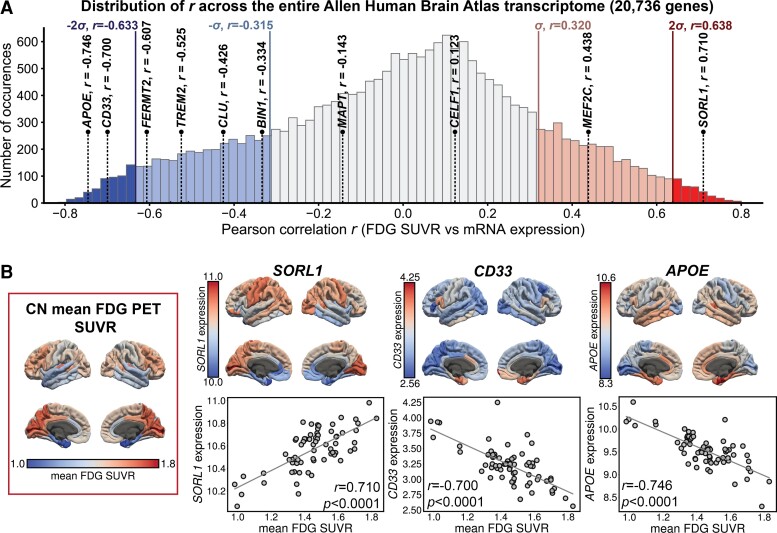
***APOE*, *CD33* and *SORL1* showed especially strong correlation with brain metabolism.** (**A**) Distribution of 20 736 Pearson correlation coefficients, calculated between expression of 20 736 genes and mean FDG PET SUVR averaged across all CN participants. Light blue and red vertical lines represent *z*-score = –1 and 1 (i.e. one standard deviation away from mean), respectively. Vertical lines labelled with σ indicate the z-score = −1 and 1 (i.e. one standard deviation away from mean), respectively. Vertical lines labelled with 2σ indicate z-score = −2 and 2. Bars in the distribution outside of the two standard deviations are coloured deep blue (negative correlations) and deep red (positive correlations), respectively. Bars in the distribution between one and two standard deviations are coloured light blue and light red, respectively. Using a *z*-score cut-off of ±2.0, *SORL1, CD33* and *APOE* showed strong correlation when compared with the distribution. (**B**) Left, in rectangular frame: mean FDG PET SUVR across all CN participants mapped to the 68 cortical ROIs. Right, top panel: expression of *SORL1, CD33* and *APOE*, mapped to the 68 cortical ROIs, respectively. Right, bottom panel: scatter plots showing the correlation between mean FDG PET SUVR and gene expression of *SORL1*, *CD33* and *APOE*, respectively. A linear fit is also provided in each scatter plot. FDG SUVR for each cohort and for each gender can be found in Supplementary Tables 16–19.

To gauge the significance of the correlation for the 10 Alzheimer-related genes, as compared with all 20 736 genes available in the AHBA, the correlation between the expression of the 20 736 genes and mean FDG PET SUVR of all CN participants was calculated. The resulting 20 736 correlation coefficients formed a bell-like distribution (Fig. 4A). Most Alzheimer-related genes’ correlations with brain metabolism were at least one standard deviation away from the mean of the distribution. Among them, *SORL1* (*z* = 2.228, *p_e_* = 0.0130), *CD33* (*z* = –2.208, *p_e_* = 0.0136) and *APOE* (*z* = –2.353, *p_e_* = 0.0093) were more than two standard deviations above/below the mean (|*z*| > 2), showing exceptionally strong correlation. The *z*-scores and *p_e_* for all Alzheimer genes studied can be found in Supplementary Table 2.

Brain regions with high *APOE* and *CD33* expression had low FDG PET SUVR, and regions with high *SORL1* expression had high FDG PET SUVR (Fig. 4B). *APOE* and *CD33* expressions were especially high (and *SORL1* expression and FDG PET SUVR were especially low) in regions most susceptible to Alzheimer’s disease, such as para-hippocampus and entorhinal cortex. For *APOE*, *CD33* and *SORL1*, scatter plots between brain metabolism and gene expression exhibited strong linearity (Fig. 4B). Maps of mRNA expression for all genes studied and their correlations with regional metabolism are provided in Supplementary Fig. 4. Overall, most genes exhibited a strong linear relationship with brain metabolism. For genes whose expression was positively correlated with FDG PET SUVR, their expression in Alzheimer’s-disease-related regions were lower. Contrarily, for genes whose expression was negatively correlated with FDG PET SUVR, their expression in Alzheimer’s-disease-related regions were higher.

### Correlation between brain gene expression and metabolism by modules

**Figure 5:**
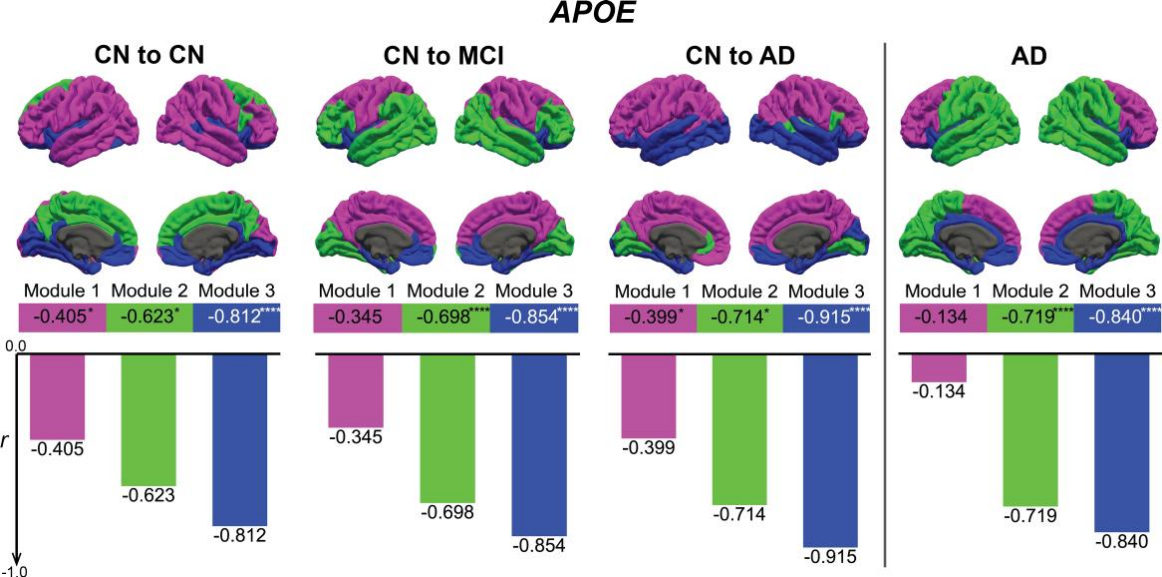
**Module-wise correlation between *APOE* expression and brain metabolism.** The four columns, from left to right, show on the top the four aspects of the brain with the modular regions calculated for ‘CN to CN’, ‘CN to MCI’, ‘CN to AD’ and ‘AD’ group, respectively. For each group, in the lower section of the figure, the Pearson correlation (*r*) between gene expression and brain glucose metabolism is shown for each module. Module definition is identical to Fig. 2C. Due to the lack of gene expression data for subcortical regions, the correlations were only calculated on the 68 cortical regions. Significance level of *r* is defined as: *P ≤ 0.05, **P ≤ 0.01, ***P ≤ 0.001, ****P ≤ 0.0001.

Metabolism in the Louvain algorithm-derived module containing regions related to Alzheimer’s disease drove the strong correlation between *APOE* expression and brain metabolism (Fig. 5, Module 3). *APOE* expression in Module 1 showed the weakest correlation with brain metabolism while the correlation for Module 2 was lower than for Module 3 but remained significant. For the remaining genes, while specific correlations varied with the gene and the participant group under question, Module 2 and Module 3 frequently exhibited a stronger correlation, whereas Module 1 exhibited a weaker correlation (Supplementary Fig. 5).

## Discussion

Our work yielded two main original findings: (i) metabolic brain networks are progressively disrupted as the risk for developing Alzheimer’s disease increases; (ii) particularly in areas of the brain prone to be affected by Alzheimer’s disease there was a correlation between risk-gene expression and metabolism.

### Progressive disruption of brain metabolic networks as risk for Alzheimer’s disease increases

We detected a monotonic increase in modularity of brain metabolic networks in four groups of elderly participants with increasing risk for Alzheimer’s disease: ‘CN to CN’, ‘CN to MCI’, ‘CN to AD’ and ‘AD’ (Fig. 2B), driven by weakened connections between the 72 ROIs. While the increase of FDG PET network modularity from CN controls to Alzheimer’s disease patients was previously reported,^6^ here we focused on a stratified CN cohort based on risk for Alzheimer’s disease.

The challenges faced by Alzheimer’s disease studies using FDG PET networks are two-fold. First, the interpretation of a PET network is less intuitive than a conventional functional MRI network, as correlation between regions are calculated across participants instead of time points. Second, a more continuous definition of risk for Alzheimer’s disease is needed to validate any trend of the local or global measures of network structure observed in research with binary participant classification (e.g. CN controls versus Alzheimer’s disease patients).

The present study addresses both challenges. We interpreted the correlation in FDG PET networks as metabolic co-activation. The underlying hypothesis is that for a group of participants sharing certain characteristics (e.g. participants with high risk for Alzheimer’s disease), their brain metabolic activities share a common pattern. Metabolic activity of each participant serves as a single data point observed from this pattern, much like a time point observed in the time-series data of functional MRI. The correlation across these data points describes the shared functional pattern of brain metabolism.

This hypothesis calls for a clear definition of participant groups and rigorous quality control, as any noise introduced into each group threatens to hinder the observation of a common metabolic pattern. We here stratified CN participants based on their 5-year progression (Supplementary Fig. 1) and inspected our participants with considerable care. Our stratification differs from previous studies that adopted varying follow-up lengths, usually due to the different lengths that participants stayed in the study. For instance, a CN participant at baseline that developed Alzheimer’s disease at the tenth year of follow-up is unlikely to be very different from a CN participant at baseline that stayed CN for 3 years but then left the study. However, many studies would classify the two participants into two different groups, as they had ‘different future progression’.^33,34^ Our selection of CN participants avoided the ambiguity brought by varying follow-up lengths.

While within-module connections dropped slightly from the ‘CN to CN’ group to the ‘AD’ group, it was the drastic decrease in between-module connections that raised modularity. Such changes are linked to the concept of ‘network fragmentation’,^35^ which refers to the splitting of an integrated network into poorly connected modules, usually due to a substantial loss of connectivity between brain regions or a targeted attack against hub nodes. The fragmentation is speculated to cause a lack of communication between brain regions and thus interrupt the integrated function of the system. We argue that modularity is an accurate measure of the fragmentation of brain metabolic network, and the increased modularity in our participants at risk for Alzheimer’s disease aligns well with the notion that the human brain goes through network failure as Alzheimer’s disease progresses.^36^

The 5-year follow-up chosen in the present study is longer than for many comparable studies,^34,37^ and while other studies followed some participants for a longer period of time, the varying follow-up lengths made interpretation difficult.^33,34^ On the other hand, our strict definition of risk for Alzheimer’s disease led to limited sample sizes, especially in the ‘CN to MCI’ and ‘CN to AD’ groups. Future efforts in expanding sample size under similar group definition are crucial to validate the results presented here.

### The effect of sex on metabolic networks of participants at risk for Alzheimer’s disease

The level of network fragmentation differed by sex (Fig. 2D and E and Supplementary Fig. 2). For ‘CN to CN’, ‘CN to AD’ and ‘AD’ groups, women consistently exhibited more modular metabolic patterns and weaker overall connection strength than men. Two-tailed *t*-test found no significant differences in age between men and women in all but the ‘AD’ group where men were significantly older than women.

Previous studies reported that women are at higher risk for Alzheimer’s disease than men^38^ and neurodegeneration and clinical symptoms may evolve more rapidly in women once a diagnosis is suspected.^39^ While a longer life expectancy of women might be the reason for the sex-specific risk for Alzheimer’s disease, increasing evidence argues for neurobiological differences between the sexes.^40^ One such sex difference may be a diverse metabolic network strength with aging, as determined by the present study. The small sample sizes of ‘CN to MCI’ and ‘CN to AD’ groups limited the generalization of our finding, and the same analysis should be repeated in larger samples. Further research is needed to pinpoint the exact cause of the difference in modularity and connection strengths between men and women. Furthermore, it should be stressed that we studied older individuals; FDG PET network modularity may be different in younger samples.

### Relationship between regional brain expression of Alzheimer-related genes and brain metabolism

The brain expression of 8 of the 10 Alzheimer-related genes had a significant correlation with brain metabolism across all four groups, and among them *SORL1*, *FERMT2*, *CD33* and *APOE* showed a stronger absolute correlation with brain metabolism as risk for Alzheimer’s disease increased in CN participants (Fig. 3). Four of the ten genes are related to the immune system: *CD33*, *CLU*, *MEF2C* and *TREM2*. Contrasting gene correlation values against a null-hypothesis distribution revealed three genes with exceptionally strong correlation with brain metabolism (Fig. 4): *APOE* (*r* = –0.746), *SORL1* (*r* = 0.710) and *CD33* (*r* = –0.700).

Apolipoprotein E (*APOE*) genotype is well-known to be related to late onset Alzheimer’s disease. Its isoform, *APOE* ε4, is the strongest genetic risk factor for Alzheimer’s disease.^41^ Though most studies focused on isoform-specific differences in structure and function, the main function of the ApoE protein, the redistribution of lipoproteins and cholesterol, is not sufficient to explain *APOE*’s detrimental effect in Alzheimer’s disease. Consequently, studying the mRNA expression of *APOE* could potentially provide a new perspective on the understanding of the pathology of Alzheimer’s disease. Several post-mortem brain studies reported elevated RNA expression of *APOE* in Alzheimer’s disease patients regardless of *APOE* genotype.^11,42^ In *APOE* ε3/ε3 human brain, *APOE*-mRNA levels were significantly increased in brains affected by Alzheimer’s disease compared with controls.^11^ Concordantly, we demonstrated that *APOE* had a strong negative correlation with brain glucose metabolism in an older population (Fig. 3). By splitting participants into 3/3, 3/4 and 4/4 genotypes, we found that such strong negative correlation did not depend on the participants’ *APOE* genotype (Supplementary Fig. 3). Granted, the mRNA expression data used in the present work are from the AHBA, and the donor’s *APOE* genotype was not collected. Ideally, mRNA expression and imaging data should be collected from the same participants.

Sortilin related receptor 1 gene (*SORL1*) showed the strongest positive correlation with brain metabolism across all four groups. *SORL1* encodes a mosaic protein of the low-density lipoprotein receptor family. Scherzer *et al.*^43^ suggested that *SORL1* interacts with *APOE* as an encoder of the mosaic ApoE receptor. They observed a significant reduction in *SORL1* expression in brain tissue of Alzheimer’s disease patients, postulating a protective effect of *SORL1*. Our results support this postulate, as we observed the strongest positive correlation between *SORL1* mRNA expression and brain glucose metabolism, opposite the direction of correlation between *APOE* and metabolism.

CD33 is a sialic acid-binding immunoglobulin-like lectin that regulates innate immunity. In the brain, *CD33* is mainly expressed in microglial cells. In a study that involved both *CD33* knockout mice and human brain samples, the density of *CD33*-immunoreactive microglia positively correlated with Aβ burden and Alzheimer’s disease patients had a 5-fold increase in *CD33* mRNA relative to controls.^10^ Another study reported that the risk allele is associated with a 7-fold increase in *CD33* cell surface expression of circulating monocytes.^44^ The strong negative correlation between *CD33* expression and FDG PET SUVR observed here (Fig. 3) echoed the finding that elevated *CD33* expression may increase risk for Alzheimer’s disease. Higher *CD33* expression corresponds to hypometabolism in the brain, which is associated with Alzheimer’s disease, though no causal relationship can be derived from the correlation.

The prevalence of immunity-related genes identified in our analysis is consistent with recent genome-wide association studies (GWAS) indicating that many risk genes for Alzheimer’s disease are part of the innate immune response pathways,^45,46^ which has led to a growing branch of Alzheimer’s disease research focusing on neuroinflammation.^47^ The strong correlations observed between immune system-related gene expression and FDG PET SUVR suggest that these genes may contribute to risk for Alzheimer’s disease through interaction between immune response and metabolism. Metabolic processes regulate immune cell responses, and inappropriate immune activation can dysregulate cellular metabolism.^20^ Several groups have studied Alzheimer’s disease in the context of immunometabolism and suggested that defects in energy metabolisms caused microglia dysfunction in the disease.^20,48^  *MEF2C* and *TREM2* are associated with immunometabolism.^49,50^ The gene correlation observed in the present study supports the speculation that immune response and brain metabolism interact with each other through a set of risk genes for Alzheimer’s disease, contributing to disease development. However, only Ulland *et al.*^50^ observed an immunometabolic interaction in post-mortem human brain, and how such interaction leads to the correlation observed in the current work remains unclear.

It is important to note that the correlations between gene expression and metabolism we report here were observed in an older, cognitively unimpaired, population and in people with Alzheimer’s disease. It is possible that they may have been quite different in a younger sample. The correlations in older age may reflect the additive metabolic impact through the years of the genetic makeup of the people included in our sample. Some features of Alzheimer’s disease are similar to those of normal aging^51^ and age is the strongest risk factor for the development of Alzheimer’s disease.^52^

The above observations should be validated with gene expression and imaging data obtained in the same participants. Due to a lack of gene expression data in the ADNI database, the present study could not take into consideration the change of gene expression as risk for Alzheimer’s disease increased. Further, what drives the differential expression of these genes across the brain^8^ is a question beyond the scope of the current study.

### The strongest correlation between gene expression and metabolism was in Alzheimer-related regions and particularly metabolic Module 3

Alzheimer’s disease does not affect all the brain uniformly, but some regions are well-known to be affected earlier and more profoundly.^2^ These regions largely coincided with Module 3 (Fig. 2C), identified by the data-driven, modular partition of the brain metabolic networks, confirming that modularity maximization leads to functionally meaningful partitions. Furthermore, for most AD-related genes, metabolism in regions related to Alzheimer’s disease drove the strong correlation between gene expression and FDG PET SUVR (Fig. 5 and Supplementary Fig. 5). For *APOE*, the correlation was strongest in Module 3, which contained regions most susceptible to Alzheimer’s disease, particularly the anteromedial temporal region.^53^ Such correlation was second strongest in Module 2, which contained regions affected by Alzheimer’s disease, but usually at a later stage in disease development. The specific composition of this module varied considerably with risk groups. Module 1, on the contrary, contained regions largely unaffected by Alzheimer’s disease pathology, and showed very weak correlation between *APOE* expression and brain glucose metabolism. *SORL1* showed largely the same pattern (Supplementary Fig. 5).

While others have investigated differential expression of *APOE*^54^ and *SORL1*^55^ between brain regions susceptible to Alzheimer’s disease and brain regions resistant to Alzheimer’s disease, evidence presented here point out that these genes may contribute to the regional vulnerability of human brain to Alzheimer’s disease pathology^56^ through interaction with brain metabolism. Unique to *APOE*, the gene’s correlation with brain glucose metabolism monotonically increased from ‘CN to CN’ to ‘CN to AD’ group for both Module 2 and Module 3 (Fig. 5). We speculate that the correlation between *APOE* gene expression and brain metabolism, especially among regions most affected by Alzheimer’s disease, could be another indicator of risk for the disease. However, although the correlation between regional metabolism and the expression of the genes conferring the highest risk was strongest in participants with the highest risk, even those with the lowest risk showed a high correlation in Module 3. This commonality could be related to older age, shared by all participants. If this is the case, younger individuals may show a different pattern. This analysis would further clarify the relationship of regional metabolism and gene expression across the lifespan.

## Conclusions

Evidence presented here shows that modularity of the human brain metabolic network can serve as an indicator of the level of dysfunction caused by network fragmentation, and that larger modularity correlates with higher risk for Alzheimer’s disease among CN individuals. Unprecedentedly, the brain expression of most Alzheimer-related genes was shown to significantly correlate with regional brain metabolism across all risk groups, with *APOE* showing the strongest negative correlation and *SORL1* showing the strongest positive correlation, particularly in the metabolic module including brain regions earliest affected in the disease. These novel results emphasize the importance of brain metabolism in potentially mediating the effect of Alzheimer’s risk genes.

## Supplementary Material

fcac216_Supplementary_Data

## Abbreviations

ADAS11 =: Alzheimer’s disease assessment Scale 11 tasks
ADAS13 =: Alzheimer’s disease assessment Scale 13 tasks
ADNI =: Alzheimer’s Disease Neuroimaging Initiative
AHBA =: Allen Human Brain Atlas
CDR-SB =: clinical dementia rating sum of boxes
CN =: cognitively normal
FDG =: fluorodeoxyglucose
MCI =: mild cognitive impairment
MMSE =: mini-mental state examination
MOCA =: Montreal Cognitive Assessment
ROI =: region of interest
SUVR =: standardized uptake value ratio
