# Supplementary material for: In Alzheimer-prone brain regions, metabolism and risk-gene expression are strongly correlated
Source: Brain Commun. 2022 Aug 25;4(5):fcac216. doi: 10.1093/braincomms/fcac216 (PMC9453434; doi:10.1093/braincomms/fcac216)
Supplement: fcac216_Supplementary_Data [file fcac216_supplementary_data.docx]

**Metabolism predicts Alzheimer risk on network level and is strongly linked to risk-gene expression on region level**

Fengdan Ye, Quentin Funk, Elijah Rockers, Joshua M. Shulman, Joseph C. Masdeu, Belen Pascual, for the Alzheimer’s Disease Neuroimaging Initiative

Table of Contents

[Supplementary Tables 3](#_Toc110939464)

[**Supplementary Table 1.** *P*-values of test for significant differences of demographics between groups. 3](#_Toc110939465)

[**Supplementary Table 2.** *Z*-scores and the corresponding effective *p*-values (*p*_e_) for all 10 AD-related genes. 3](#_Toc110939466)

[Supplementary Figures 4](#_Toc110939467)

[**Supplementary Figure 1.** Illustration of the data processing pipeline. 4](#_Toc110939468)

[**Supplementary Figure 2.** Effect of sex on the metabolic networks. 6](#_Toc110939469)

[**Supplementary Figure 3.** The effect of *APOE* genotype on the correlation between gene expression and brain glucose metabolism. 6](#_Toc110939470)

[**Supplementary Figure 4.** Mapping of mean FDG PET SUVR and gene expression to brain. 7](#_Toc110939471)

[**Supplementary Figure 5.** Module-wise correlation between expression of 10 AD-related genes and brain metabolism. 9](#_Toc110939472)

[Appendix A: List of ROIs in Correlation Matrices 10](#_Toc110939473)

[Original list of 72 brain regions 10](#_Toc110939474)

[**Supplementary Table 3.** Ordered list of ROIs for original correlation matrices. 10](#_Toc110939475)

[Partitioned List of 72 Brain Regions 11](#_Toc110939476)

[CN to CN 11](#_Toc110939477)

[**Supplementary Table 4.** Ordered list of ROIs for the clustered correlation matrix of “CN to CN” group (Figure 2B, first column). 12](#_Toc110939478)

[**Supplementary Table 5.** Ordered list of ROIs for the clustered correlation matrix of “CN to CN” group - women only (Supplementary Figure 2B, first column). 13](#_Toc110939479)

[**Supplementary Table 6.** Ordered list of ROIs for the clustered correlation matrix of “CN to CN” group - men only (Supplementary Figure 2E, first column). 15](#_Toc110939480)

[CN to MCI 17](#_Toc110939481)

[**Supplementary Table 7.** Ordered list of ROIs for the clustered correlation matrix of “CN to MCI” group (Figure 2B, second column). 17](#_Toc110939482)

[**Supplementary Table 8.** Ordered list of ROIs for the clustered correlation matrix of “CN to MCI” group - women only (Supplementary Figure 2B, second column). 18](#_Toc110939483)

[**Supplementary Table 9.** Ordered list of ROIs for the clustered correlation matrix of “CN to MCI” group - men only (Supplementary Figure 2E, second column). 20](#_Toc110939484)

[CN to AD 21](#_Toc110939485)

[**Supplementary Table 10.** Ordered list of ROIs for the clustered correlation matrix of “CN to AD” group (Figure 2B, third column). 22](#_Toc110939486)

[**Supplementary Table 11.** Ordered list of ROIs for the clustered correlation matrix of “CN to AD” group - women only (Supplementary Figure 2B, third column). 23](#_Toc110939487)

[**Supplementary Table 12.** Ordered list of ROIs for the clustered correlation matrix of “CN to AD” group - men only (Supplementary Figure 2E, third column). 25](#_Toc110939488)

[AD 26](#_Toc110939489)

[**Supplementary Table 13.** Ordered list of ROIs for the clustered correlation matrix of “AD” group (Figure 2B, fourth column). 26](#_Toc110939490)

[**Supplementary Table 14.** Ordered list of ROIs for the clustered correlation matrix of “AD” group - women only (Supplementary Figure 2B, fourth column). 28](#_Toc110939491)

[**Supplementary Table 15.** Ordered list of ROIs for the clustered correlation matrix of “AD” group - men only (Supplementary Figure 2E, fourth column). 30](#_Toc110939492)

[Appendix B: Mean and Standard Deviation of FDG SUVR 32](#_Toc110939493)

[CN to CN 32](#_Toc110939494)

[**Supplementary Table 16.** Mean and standard deviation of FDG SUVR within the “CN to CN” group. 32](#_Toc110939495)

[CN to MCI 34](#_Toc110939496)

[**Supplementary Table 17.** Mean and standard deviation of FDG SUVR within the “CN to MCI” group. 34](#_Toc110939497)

[CN to AD 36](#_Toc110939498)

[**Supplementary Table 18.** Mean and standard deviation of FDG SUVR within the “CN to AD” group. 36](#_Toc110939499)

[AD 38](#_Toc110939500)

[**Supplementary Table 19.** Mean and standard deviation of FDG SUVR within the “AD” group. 38](#_Toc110939501)

# Supplementary Tables

## **Supplementary Table 1.** *P*-values of test for significant differences of demographics between groups.

|  | **Sex** | **Age** | **Age (Men)** | **Age (Women)** | **Years of Education** |
| --- | --- | --- | --- | --- | --- |
| **CN to CN vs CN to MCI** | 0.977 | 0.929 | 0.806 | 0.852 | 0.199 |
| **CN to CN vs CN to AD** | 0.469 | 0.996 | 0.661 | 0.661 | 0.908 |
| **CN to CN vs AD** | 0.486 | **<0.001** | **0.001** | **0.000** | **0.002** |
| **CN to MCI vs CN to AD** | 0.535 | 0.960 | 0.792 | 0.779 | 0.502 |
| **CN to MCI vs AD** | 0.658 | **<0.001** | **0.021** | **<0.001** | 0.371 |
| **CN to AD vs AD** | 0.694 | **0.001** | 0.202 | **0.002** | 0.204 |

Sex: two-tailed *z*-test for proportions. Age and years of education: Two-sample two-tailed *t*-tests with unequal variance. *P*-values smaller than 0.05 are labelled in bold. AD = Alzheimer’s disease; CN = Cognitively normal; MCI = Mild cognitive impairment.

## **Supplementary Table 2.** *Z*-scores and the corresponding effective *p*-values (*p*_e_) for all 10 AD-related genes.

| **Gene** | **All CN** | | **CN to CN** | | **CN to MCI** | | **CN to AD** | | **AD** | |
| --- | --- | --- | --- | --- | --- | --- | --- | --- | --- | --- |
|  | ***z*** | ***p*_e_** | ***z*** | ***p*_e_** | ***z*** | ***p*_e_** | ***z*** | ***p*_e_** | ***z*** | ***p*_e_** |
| ***SORL1*** | 2.228 | 0.013 | 2.219 | 0.013 | 2.229 | 0.013 | 2.253 | 0.012 | 2.228 | 0.013 |
| ***MEF2C*** | 1.371 | 0.085 | 1.384 | 0.083 | 1.397 | 0.081 | 1.266 | 0.103 | 1.474 | 0.070 |
| ***CELF1*** | 0.378 | 0.353 | 0.376 | 0.354 | 0.411 | 0.341 | 0.343 | 0.366 | 0.332 | 0.370 |
| ***MAPT*** | -0.458 | 0.324 | -0.441 | 0.330 | -0.533 | 0.297 | -0.436 | 0.332 | -0.726 | 0.234 |
| ***BIN1*** | -1.058 | 0.145 | -1.038 | 0.150 | -1.157 | 0.124 | -1.017 | 0.155 | -1.359 | 0.087 |
| ***CLU*** | -1.347 | 0.089 | -1.331 | 0.092 | -1.409 | 0.080 | -1.333 | 0.091 | -1.349 | 0.089 |
| ***TREM2*** | -1.659 | 0.049 | -1.675 | 0.047 | -1.636 | 0.051 | -1.605 | 0.054 | -1.516 | 0.065 |
| ***FERMT2*** | -1.916 | 0.028 | -1.918 | 0.028 | -1.872 | 0.031 | -1.956 | 0.025 | -1.878 | 0.030 |
| ***CD33*** | -2.208 | 0.014 | -2.229 | 0.013 | -2.142 | 0.016 | -2.181 | 0.015 | -2.063 | 0.020 |
| ***APOE*** | -2.353 | 0.009 | -2.362 | 0.009 | -2.311 | 0.010 | -2.355 | 0.009 | -2.490 | 0.006 |

The *z*-scores were calculated from the null distribution of the correlation coefficients between the expression of the 20,736 genes and mean FDG PET SUVR. The latter was obtained from either across all CN participants, or within each group (“CN to CN”, “CN to MCI”, “CN to AD”, or “AD”), leading to each gene having five *z*-scores.

AD = Alzheimer’s disease; CN = Cognitively normal; MCI = Mild cognitive impairment. *p_e_*: the effective *p*-values calculated from the corresponding *z*-scores. *z*: *z*-scores of correlations between gene expression and FDG PET SUVR when compared to the null distribution.

# Supplementary Figures


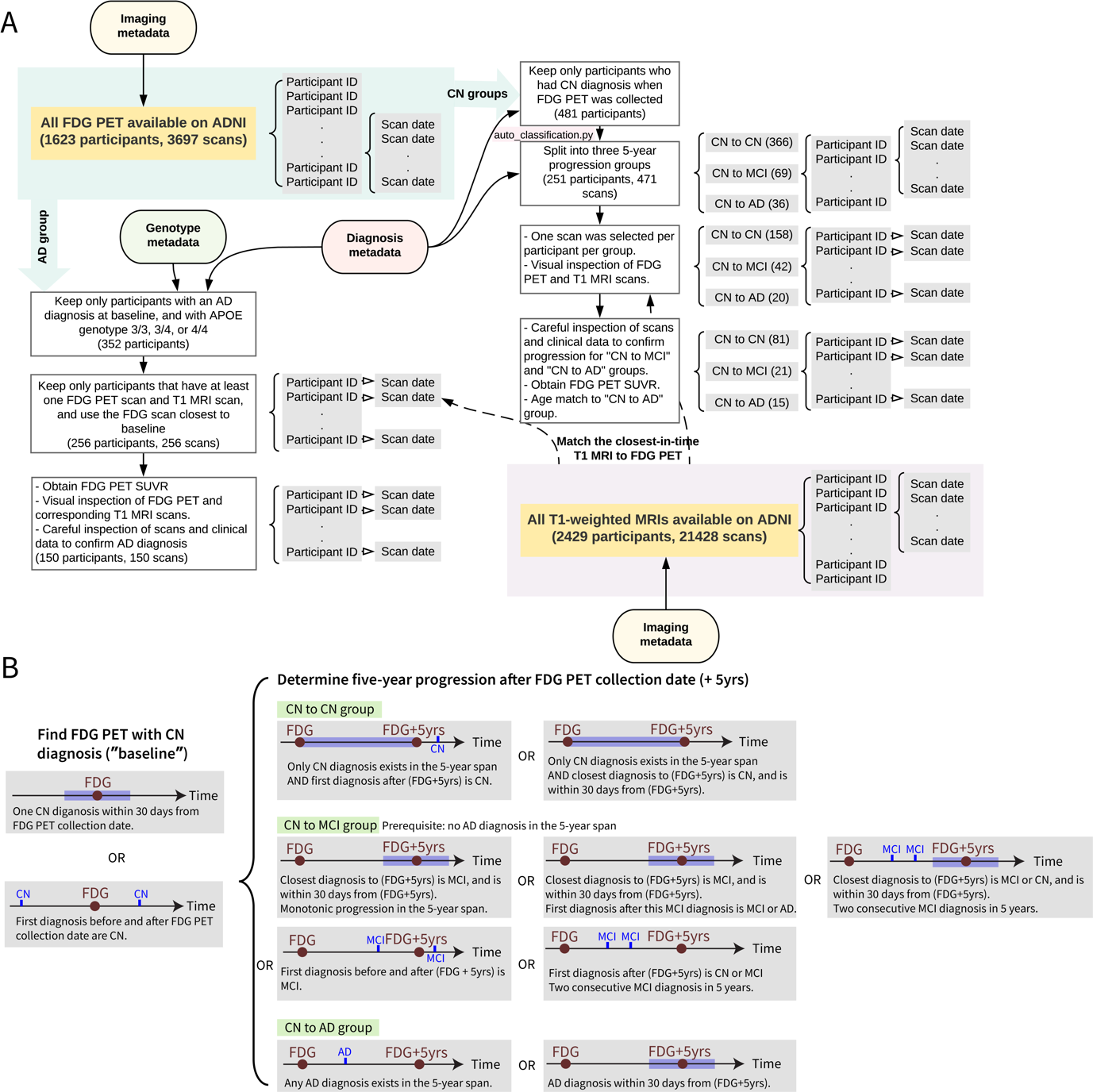


## **Supplementary Figure 1.** Illustration of the data processing pipeline.

**(A)** A diagram showing the collection, classification, and inspection of the ADNI data. In the end, there were 81 participants in the “CN to CN” group, 21 participants in the “CN to MCI” group, 15 participants in the “CN to AD” group, and 150 participants in the “AD” group. **(B)** A detailed description of grouping criteria for the three control groups. Please enlarge the pdf to read this illustration more easily.


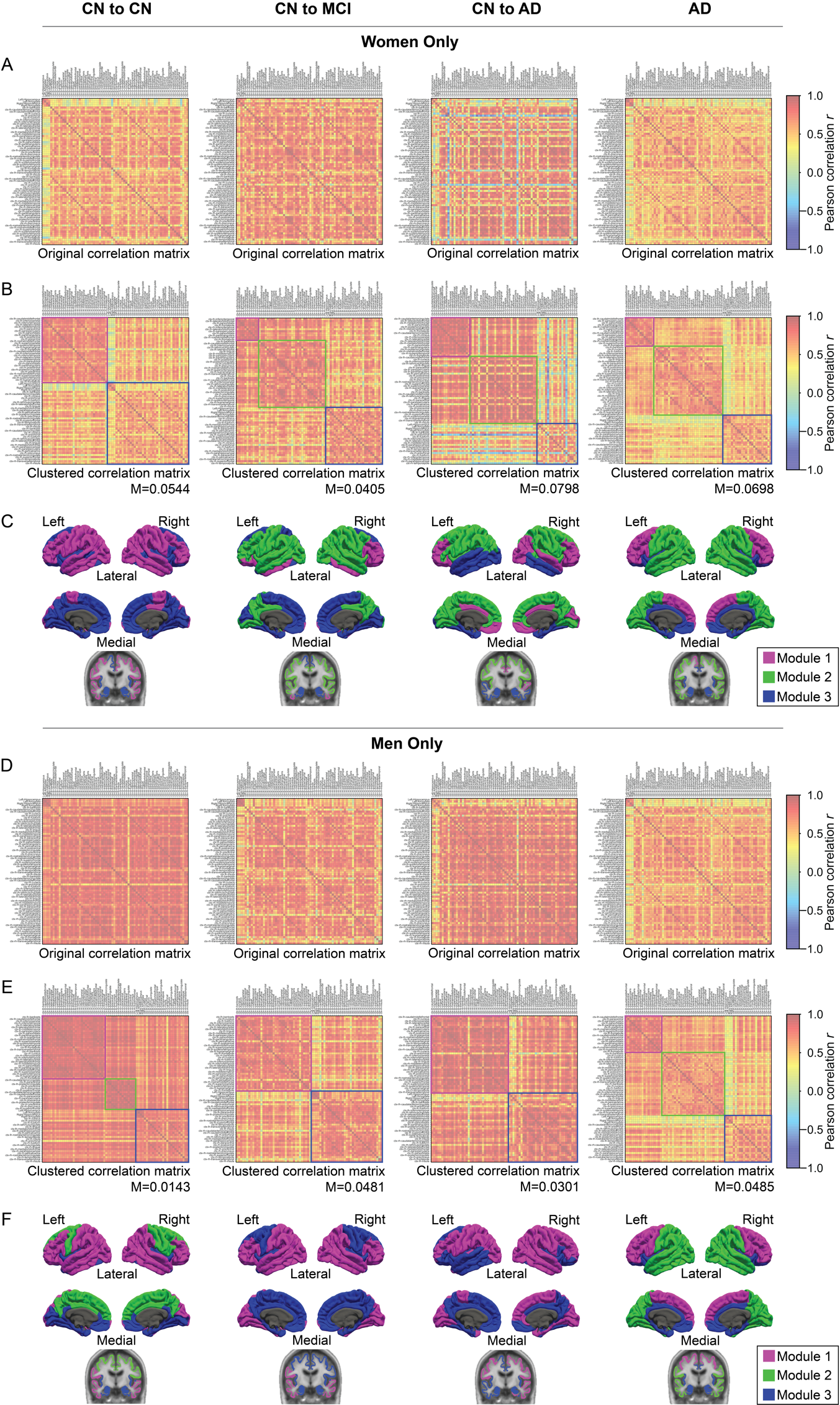


## **Supplementary Figure 2.** Effect of sex on the metabolic networks.

***Women only:*** **(A)** Original correlation matrices. Pearson correlation coefficients were calculated from mean FDG PET SUVR between 72 ROIs. These matrices fully describe the whole-brain FDG PET networks. **(B)** The re-arranged correlation matrices to reflect the clustered structure of the FDG PET networks. The colored squares represent modules. **(C)** Brain maps of modules. Each color represents one module. ***Men only:*** **(D-F)** same as (A-C) but for men only. Full list of the 72 ROIs in the matrices can be found in Supplementary Appendix.


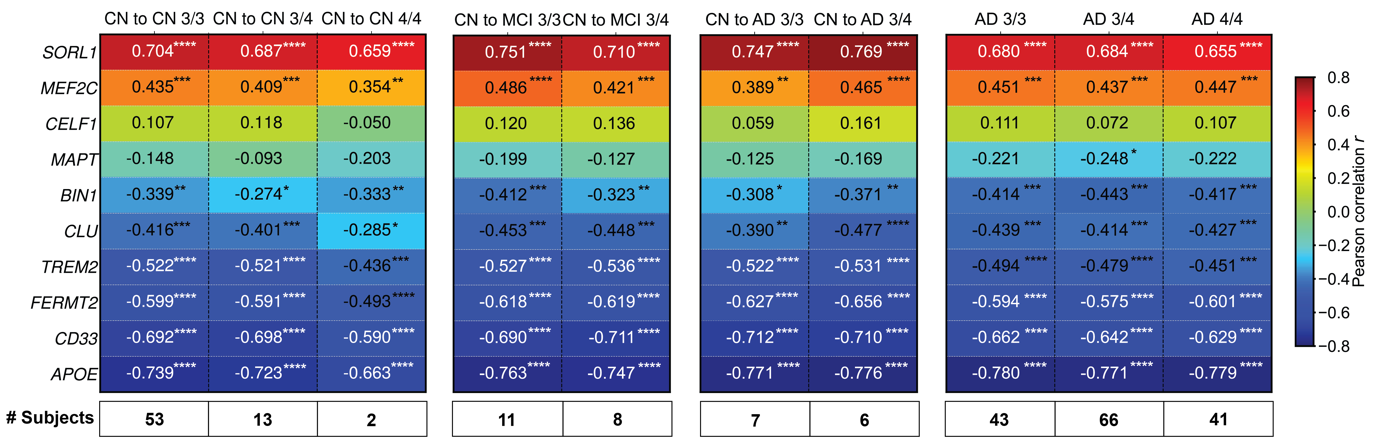


## **Supplementary Figure 3.** The effect of *APOE* genotype on the correlation between gene expression and brain glucose metabolism.

“CN to CN 3/3” means the mean FDG PET SUVR for each ROI was calculated from participants in the “CN to CN” group that have an *APOE* genotype of 3/3. The mean FDG PET SUVR profile was then correlated with gene expression to produce the column of ***r*** values under “CN to CN 3/3”. Only participants with *APOE* genotype of 3/3, 3/4, or 4/4 were considered. Significance level is defined as: *: *p*<=0.05, **: *p*<=0.01, ***: *p*<=0.001, ****: *p*<=0.0001.


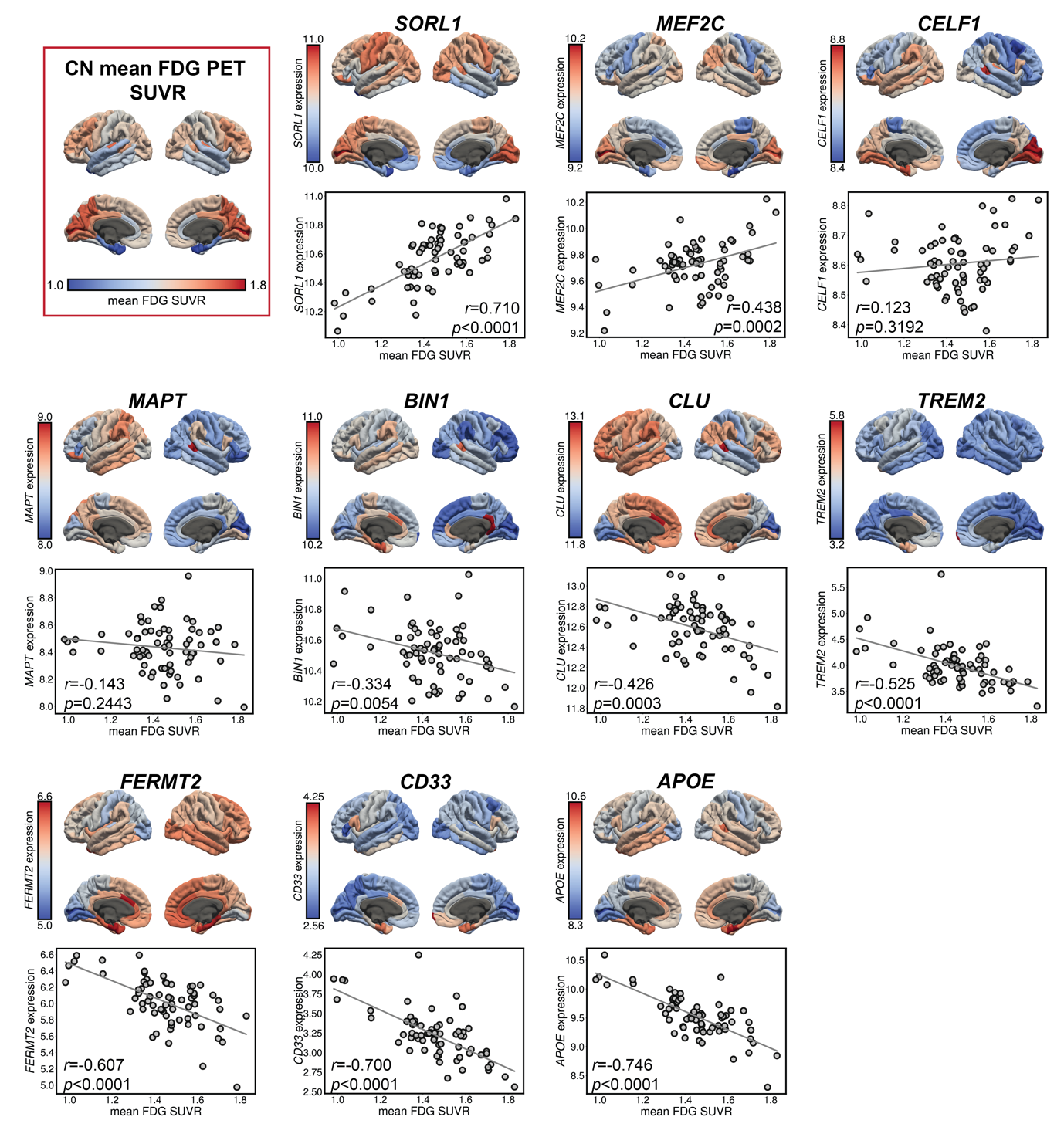


## **Supplementary Figure 4.** Mapping of mean FDG PET SUVR and gene expression to brain.

Top left, in red rectangular frame: mean FDG PET SUVR across all CN participants mapped to the 68 cortical ROIs. The rest: gene expression of all 10 AD-related genes, mapped to the 68 cortical ROIs, respectively. Under each gene expression brain map is a scatter plot showing the correlation between mean FDG PET SUVR and gene expression. A linear fit is also provided in each scatter plot.

**
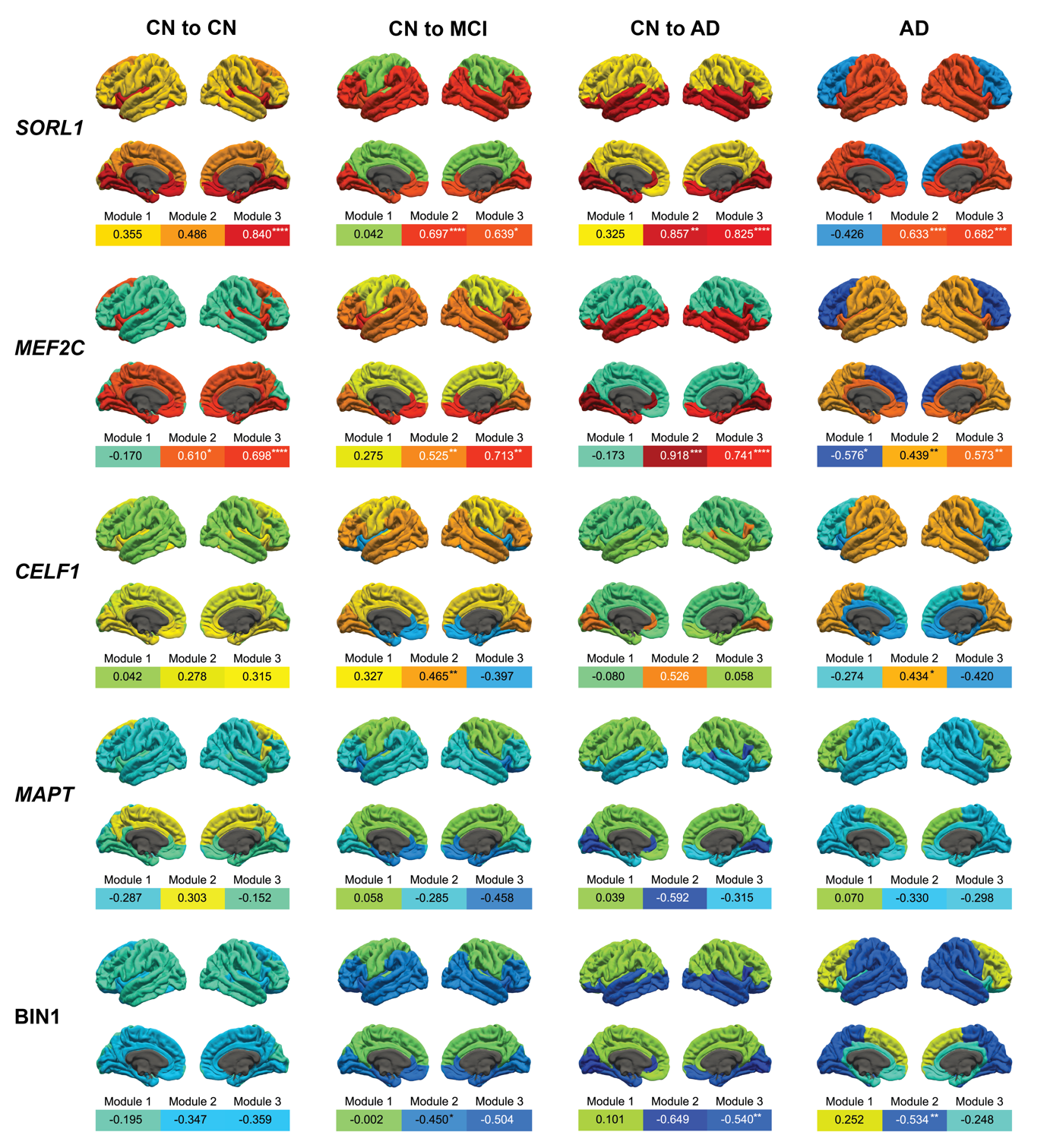
**

**
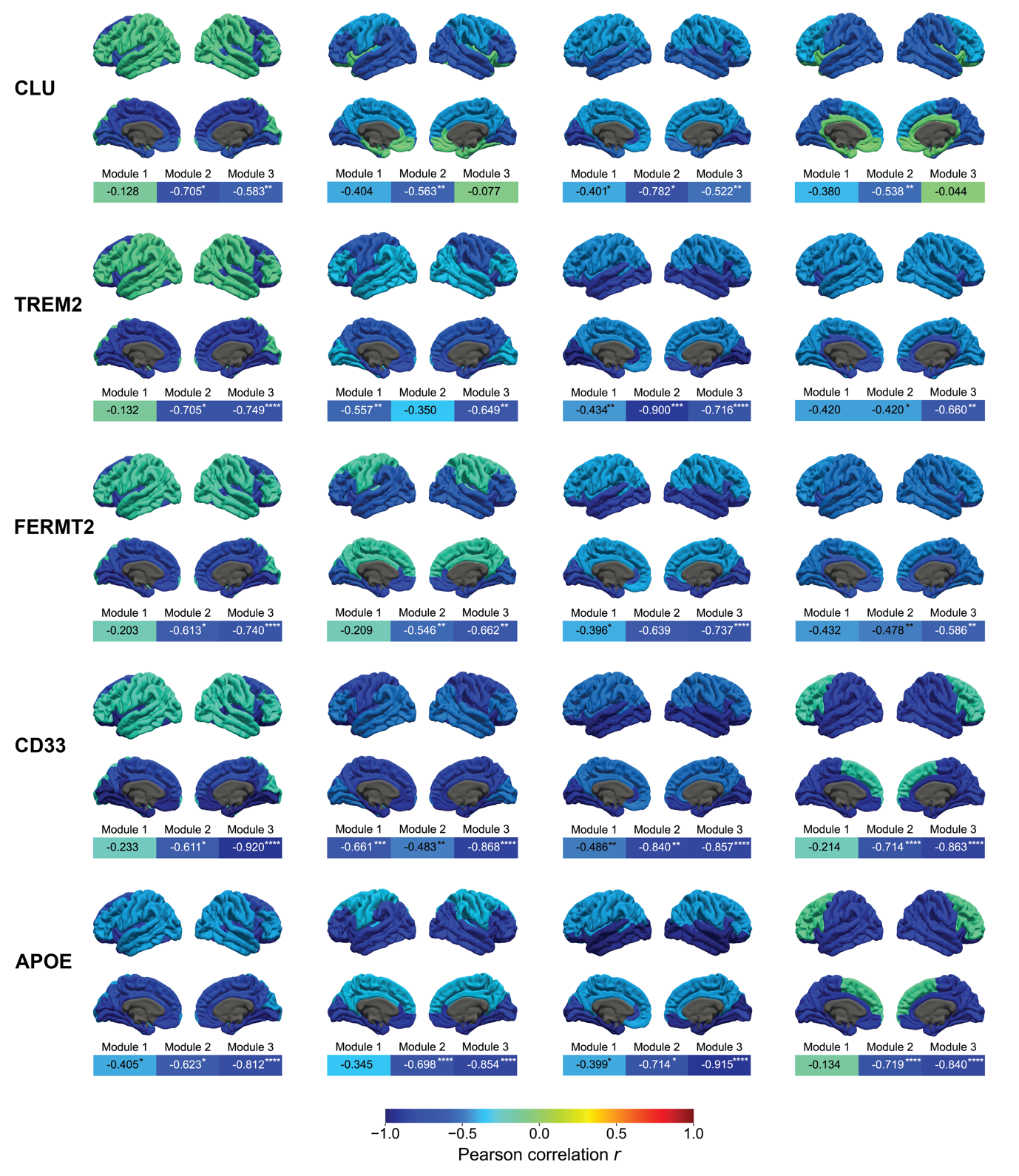
**

## **Supplementary Figure 5.** Module-wise correlation between expression of 10 AD-related genes and brain metabolism.

The four columns, from left to right, correspond to “CN to CN”, “CN to MCI”, “CN to AD”, and “AD” group, respectively. For each group, correlation (*r*) between gene expression and glucose metabolism was calculated for each module. Module location and extent are as depicted in Figure 2C, but here modules are colored based on the correlation (*r*) within each module, and a colorbar is provided at the very bottom of this figure. Red represents strong positive correlation, blue represents strong negative correlation, and green represents near-zero correlation. Please enlarge the pdf to peruse a subset of genes. Significance level is defined as: *: *p*<=0.05, **: *p*<=0.01, ***: *p*<=0.001, ****: *p*<=0.0001.

# Appendix A: List of ROIs in Correlation Matrices

### Original list of 72 brain regions

In the original correlation matrices shown in Figure 2 and Figure S2, the 72 ROIs started with Left-Hippocampus, Left-Amygdala, Right-Hippocampus, and Right-Amygdala, followed by 34 DK cortical regions on the left hemisphere (“lh-”) and then 34 DK cortical regions on the right hemisphere (“rh-”). The exact ordering is shown in Table A1. For all lists, region #1 corresponds to the top-most row (left-most column) of the correlation matrix and region #72 corresponds to the bottom-most row (right-most column).

## **Supplementary Table 3.** Ordered list of ROIs for original correlation matrices.

| **Region #** | **Region Name** |
| --- | --- |
| 1 | Left-Hippocampus |
| 2 | Left-Amygdala |
| 3 | Right-Hippocampus |
| 4 | Right-Amygdala |
| 5 | ctx-lh-bankssts |
| 6 | ctx-lh-caudalanteriorcingulate |
| 7 | ctx-lh-caudalmiddlefrontal |
| 8 | ctx-lh-cuneus |
| 9 | ctx-lh-entorhinal |
| 10 | ctx-lh-fusiform |
| 11 | ctx-lh-inferiorparietal |
| 12 | ctx-lh-inferiortemporal |
| 13 | ctx-lh-isthmuscingulate |
| 14 | ctx-lh-lateraloccipital |
| 15 | ctx-lh-lateralorbitofrontal |
| 16 | ctx-lh-lingual |
| 17 | ctx-lh-medialorbitofrontal |
| 18 | ctx-lh-middletemporal |
| 19 | ctx-lh-parahippocampal |
| 20 | ctx-lh-paracentral |
| 21 | ctx-lh-parsopercularis |
| 22 | ctx-lh-parsorbitalis |
| 23 | ctx-lh-parstriangularis |
| 24 | ctx-lh-pericalcarine |
| 25 | ctx-lh-postcentral |
| 26 | ctx-lh-posteriorcingulate |
| 27 | ctx-lh-precentral |
| 28 | ctx-lh-precuneus |
| 29 | ctx-lh-rostralanteriorcingulate |
| 30 | ctx-lh-rostralmiddlefrontal |
| 31 | ctx-lh-superiorfrontal |
| 32 | ctx-lh-superiorparietal |
| 33 | ctx-lh-superiortemporal |
| 34 | ctx-lh-supramarginal |
| 35 | ctx-lh-frontalpole |
| 36 | ctx-lh-temporalpole |
| 37 | ctx-lh-transversetemporal |
| 38 | ctx-lh-insula |
| 39 | ctx-rh-bankssts |
| 40 | ctx-rh-caudalanteriorcingulate |
| 41 | ctx-rh-caudalmiddlefrontal |
| 42 | ctx-rh-cuneus |
| 43 | ctx-rh-entorhinal |
| 44 | ctx-rh-fusiform |
| 45 | ctx-rh-inferiorparietal |
| 46 | ctx-rh-inferiortemporal |
| 47 | ctx-rh-isthmuscingulate |
| 48 | ctx-rh-lateraloccipital |
| 49 | ctx-rh-lateralorbitofrontal |
| 50 | ctx-rh-lingual |
| 51 | ctx-rh-medialorbitofrontal |
| 52 | ctx-rh-middletemporal |
| 53 | ctx-rh-parahippocampal |
| 54 | ctx-rh-paracentral |
| 55 | ctx-rh-parsopercularis |
| 56 | ctx-rh-parsorbitalis |
| 57 | ctx-rh-parstriangularis |
| 58 | ctx-rh-pericalcarine |
| 59 | ctx-rh-postcentral |
| 60 | ctx-rh-posteriorcingulate |
| 61 | ctx-rh-precentral |
| 62 | ctx-rh-precuneus |
| 63 | ctx-rh-rostralanteriorcingulate |
| 64 | ctx-rh-rostralmiddlefrontal |
| 65 | ctx-rh-superiorfrontal |
| 66 | ctx-rh-superiorparietal |
| 67 | ctx-rh-superiortemporal |
| 68 | ctx-rh-supramarginal |
| 69 | ctx-rh-frontalpole |
| 70 | ctx-rh-temporalpole |
| 71 | ctx-rh-transversetemporal |
| 72 | ctx-rh-insula |

### Partitioned List of 72 Brain Regions

### CN to CN

Table A2 shows the ordered list of ROIs for the clustered correlation matrix of “CN to CN” group (Figure 2B, first column). Table A3 shows the ordered list of ROIs for the clustered correlation matrix of women in “CN to CN” group (Supplementary Figure 2B, first column). Table A4 shows the ordered list of ROIs for the clustered correlation matrix of men in “CN to CN” group (Supplementary Figure 2E, first column).

## **Supplementary Table 4.** Ordered list of ROIs for the clustered correlation matrix of “CN to CN” group (Figure 2B, first column).

| **Module #** | **Region #** | **Region Name** |
| --- | --- | --- |
| **Module 1** | 1 | ctx-lh-bankssts |
|  | 2 | ctx-lh-caudalmiddlefrontal |
|  | 3 | ctx-lh-inferiorparietal |
|  | 4 | ctx-lh-inferiortemporal |
|  | 5 | ctx-lh-lateraloccipital |
|  | 6 | ctx-lh-middletemporal |
|  | 7 | ctx-lh-parsopercularis |
|  | 8 | ctx-lh-parsorbitalis |
|  | 9 | ctx-lh-parstriangularis |
|  | 10 | ctx-lh-postcentral |
|  | 11 | ctx-lh-precentral |
|  | 12 | ctx-lh-rostralmiddlefrontal |
|  | 13 | ctx-lh-superiorparietal |
|  | 14 | ctx-lh-superiortemporal |
|  | 15 | ctx-lh-supramarginal |
|  | 16 | ctx-lh-frontalpole |
|  | 17 | ctx-rh-cuneus |
|  | 18 | ctx-rh-inferiorparietal |
|  | 19 | ctx-rh-inferiortemporal |
|  | 20 | ctx-rh-lateraloccipital |
|  | 21 | ctx-rh-middletemporal |
|  | 22 | ctx-rh-parsorbitalis |
|  | 23 | ctx-rh-parstriangularis |
|  | 24 | ctx-rh-postcentral |
|  | 25 | ctx-rh-precentral |
|  | 26 | ctx-rh-rostralmiddlefrontal |
|  | 27 | ctx-rh-superiorparietal |
|  | 28 | ctx-rh-superiortemporal |
|  | 29 | ctx-rh-supramarginal |
|  | 30 | ctx-rh-frontalpole |
| **Module 2** | 31 | ctx-lh-caudalanteriorcingulate |
|  | 32 | ctx-lh-paracentral |
|  | 33 | ctx-lh-posteriorcingulate |
|  | 34 | ctx-lh-precuneus |
|  | 35 | ctx-lh-superiorfrontal |
|  | 36 | ctx-rh-caudalanteriorcingulate |
|  | 37 | ctx-rh-caudalmiddlefrontal |
|  | 38 | ctx-rh-paracentral |
|  | 39 | ctx-rh-parsopercularis |
|  | 40 | ctx-rh-posteriorcingulate |
|  | 41 | ctx-rh-precuneus |
|  | 42 | ctx-rh-superiorfrontal |
| **Module 3** | 43 | Left-Hippocampus |
|  | 44 | Left-Amygdala |
|  | 45 | Right-Hippocampus |
|  | 46 | Right-Amygdala |
|  | 47 | ctx-lh-cuneus |
|  | 48 | ctx-lh-entorhinal |
|  | 49 | ctx-lh-fusiform |
|  | 50 | ctx-lh-isthmuscingulate |
|  | 51 | ctx-lh-lateralorbitofrontal |
|  | 52 | ctx-lh-lingual |
|  | 53 | ctx-lh-medialorbitofrontal |
|  | 54 | ctx-lh-parahippocampal |
|  | 55 | ctx-lh-pericalcarine |
|  | 56 | ctx-lh-rostralanteriorcingulate |
|  | 57 | ctx-lh-temporalpole |
|  | 58 | ctx-lh-transversetemporal |
|  | 59 | ctx-lh-insula |
|  | 60 | ctx-rh-bankssts |
|  | 61 | ctx-rh-entorhinal |
|  | 62 | ctx-rh-fusiform |
|  | 63 | ctx-rh-isthmuscingulate |
|  | 64 | ctx-rh-lateralorbitofrontal |
|  | 65 | ctx-rh-lingual |
|  | 66 | ctx-rh-medialorbitofrontal |
|  | 67 | ctx-rh-parahippocampal |
|  | 68 | ctx-rh-pericalcarine |
|  | 69 | ctx-rh-rostralanteriorcingulate |
|  | 70 | ctx-rh-temporalpole |
|  | 71 | ctx-rh-transversetemporal |
|  | 72 | ctx-rh-insula |

## **Supplementary Table 5.** Ordered list of ROIs for the clustered correlation matrix of “CN to CN” group - women only (Supplementary Figure 2B, first column).

| **Module #** | **Region #** | **Region Name** |
| --- | --- | --- |
| **Module 1** | 1 | ctx-lh-caudalmiddlefrontal |
|  | 2 | ctx-lh-inferiorparietal |
|  | 3 | ctx-lh-inferiortemporal |
|  | 4 | ctx-lh-lateraloccipital |
|  | 5 | ctx-lh-middletemporal |
|  | 6 | ctx-lh-paracentral |
|  | 7 | ctx-lh-parsopercularis |
|  | 8 | ctx-lh-parsorbitalis |
|  | 9 | ctx-lh-parstriangularis |
|  | 10 | ctx-lh-postcentral |
|  | 11 | ctx-lh-precentral |
|  | 12 | ctx-lh-rostralmiddlefrontal |
|  | 13 | ctx-lh-superiorparietal |
|  | 14 | ctx-lh-superiortemporal |
|  | 15 | ctx-lh-supramarginal |
|  | 16 | ctx-lh-frontalpole |
|  | 17 | ctx-rh-caudalmiddlefrontal |
|  | 18 | ctx-rh-inferiorparietal |
|  | 19 | ctx-rh-inferiortemporal |
|  | 20 | ctx-rh-lateraloccipital |
|  | 21 | ctx-rh-middletemporal |
|  | 22 | ctx-rh-paracentral |
|  | 23 | ctx-rh-parsorbitalis |
|  | 24 | ctx-rh-parstriangularis |
|  | 25 | ctx-rh-postcentral |
|  | 26 | ctx-rh-posteriorcingulate |
|  | 27 | ctx-rh-precentral |
|  | 28 | ctx-rh-rostralmiddlefrontal |
|  | 29 | ctx-rh-superiorparietal |
|  | 30 | ctx-rh-superiortemporal |
|  | 31 | ctx-rh-supramarginal |
|  | 32 | ctx-rh-frontalpole |
| **Module 2** | 33 | Left-Hippocampus |
|  | 34 | Left-Amygdala |
|  | 35 | Right-Hippocampus |
|  | 36 | Right-Amygdala |
|  | 37 | ctx-lh-bankssts |
|  | 38 | ctx-lh-caudalanteriorcingulate |
|  | 39 | ctx-lh-cuneus |
|  | 40 | ctx-lh-entorhinal |
|  | 41 | ctx-lh-fusiform |
|  | 42 | ctx-lh-isthmuscingulate |
|  | 43 | ctx-lh-lateralorbitofrontal |
|  | 44 | ctx-lh-lingual |
|  | 45 | ctx-lh-medialorbitofrontal |
|  | 46 | ctx-lh-parahippocampal |
|  | 47 | ctx-lh-pericalcarine |
|  | 48 | ctx-lh-posteriorcingulate |
|  | 49 | ctx-lh-precuneus |
|  | 50 | ctx-lh-rostralanteriorcingulate |
|  | 51 | ctx-lh-superiorfrontal |
|  | 52 | ctx-lh-temporalpole |
|  | 53 | ctx-lh-transversetemporal |
|  | 54 | ctx-lh-insula |
|  | 55 | ctx-rh-bankssts |
|  | 56 | ctx-rh-caudalanteriorcingulate |
|  | 57 | ctx-rh-cuneus |
|  | 58 | ctx-rh-entorhinal |
|  | 59 | ctx-rh-fusiform |
|  | 60 | ctx-rh-isthmuscingulate |
|  | 61 | ctx-rh-lateralorbitofrontal |
|  | 62 | ctx-rh-lingual |
|  | 63 | ctx-rh-medialorbitofrontal |
|  | 64 | ctx-rh-parahippocampal |
|  | 65 | ctx-rh-parsopercularis |
|  | 66 | ctx-rh-pericalcarine |
|  | 67 | ctx-rh-precuneus |
|  | 68 | ctx-rh-rostralanteriorcingulate |
|  | 69 | ctx-rh-superiorfrontal |
|  | 70 | ctx-rh-temporalpole |
|  | 71 | ctx-rh-transversetemporal |
|  | 72 | ctx-rh-insula |

## **Supplementary Table 6.** Ordered list of ROIs for the clustered correlation matrix of “CN to CN” group - men only (Supplementary Figure 2E, first column).

| **Module #** | **Region #** | **Region Name** |
| --- | --- | --- |
| **Module 1** | 1 | ctx-lh-bankssts |
|  | 2 | ctx-lh-caudalmiddlefrontal |
|  | 3 | ctx-lh-inferiorparietal |
|  | 4 | ctx-lh-inferiortemporal |
|  | 5 | ctx-lh-lateraloccipital |
|  | 6 | ctx-lh-lateralorbitofrontal |
|  | 7 | ctx-lh-middletemporal |
|  | 8 | ctx-lh-parsopercularis |
|  | 9 | ctx-lh-parsorbitalis |
|  | 10 | ctx-lh-parstriangularis |
|  | 11 | ctx-lh-postcentral |
|  | 12 | ctx-lh-rostralmiddlefrontal |
|  | 13 | ctx-lh-superiorparietal |
|  | 14 | ctx-lh-superiortemporal |
|  | 15 | ctx-lh-supramarginal |
|  | 16 | ctx-lh-frontalpole |
|  | 17 | ctx-lh-transversetemporal |
|  | 18 | ctx-rh-bankssts |
|  | 19 | ctx-rh-cuneus |
|  | 20 | ctx-rh-inferiorparietal |
|  | 21 | ctx-rh-inferiortemporal |
|  | 22 | ctx-rh-lateraloccipital |
|  | 23 | ctx-rh-lateralorbitofrontal |
|  | 24 | ctx-rh-middletemporal |
|  | 25 | ctx-rh-parsorbitalis |
|  | 26 | ctx-rh-parstriangularis |
|  | 27 | ctx-rh-rostralmiddlefrontal |
|  | 28 | ctx-rh-superiorparietal |
|  | 29 | ctx-rh-superiortemporal |
|  | 30 | ctx-rh-supramarginal |
|  | 31 | ctx-rh-frontalpole |
| **Module 2** | 32 | ctx-lh-caudalanteriorcingulate |
|  | 33 | ctx-lh-paracentral |
|  | 34 | ctx-lh-posteriorcingulate |
|  | 35 | ctx-lh-precentral |
|  | 36 | ctx-lh-precuneus |
|  | 37 | ctx-lh-superiorfrontal |
|  | 38 | ctx-rh-caudalanteriorcingulate |
|  | 39 | ctx-rh-caudalmiddlefrontal |
|  | 40 | ctx-rh-paracentral |
|  | 41 | ctx-rh-parsopercularis |
|  | 42 | ctx-rh-postcentral |
|  | 43 | ctx-rh-posteriorcingulate |
|  | 44 | ctx-rh-precentral |
|  | 45 | ctx-rh-precuneus |
|  | 46 | ctx-rh-superiorfrontal |
| **Module 3** | 47 | Left-Hippocampus |
|  | 48 | Left-Amygdala |
|  | 49 | Right-Hippocampus |
|  | 50 | Right-Amygdala |
|  | 51 | ctx-lh-cuneus |
|  | 52 | ctx-lh-entorhinal |
|  | 53 | ctx-lh-fusiform |
|  | 54 | ctx-lh-isthmuscingulate |
|  | 55 | ctx-lh-lingual |
|  | 56 | ctx-lh-medialorbitofrontal |
|  | 57 | ctx-lh-parahippocampal |
|  | 58 | ctx-lh-pericalcarine |
|  | 59 | ctx-lh-rostralanteriorcingulate |
|  | 60 | ctx-lh-temporalpole |
|  | 61 | ctx-lh-insula |
|  | 62 | ctx-rh-entorhinal |
|  | 63 | ctx-rh-fusiform |
|  | 64 | ctx-rh-isthmuscingulate |
|  | 65 | ctx-rh-lingual |
|  | 66 | ctx-rh-medialorbitofrontal |
|  | 67 | ctx-rh-parahippocampal |
|  | 68 | ctx-rh-pericalcarine |
|  | 69 | ctx-rh-rostralanteriorcingulate |
|  | 70 | ctx-rh-temporalpole |
|  | 71 | ctx-rh-transversetemporal |
|  | 72 | ctx-rh-insula |

### CN to MCI

Table A5 shows the ordered list of ROIs for the clustered correlation matrix of “CN to MCI” group (Figure 2B, second column). Table A6 shows the ordered list of ROIs for the clustered correlation matrix of women in “CN to MCI” group (Supplementary Figure 2B, second column). Table A7 shows the ordered list of ROIs for the clustered correlation matrix of men in “CN to MCI” group (Supplementary Figure 2E, second column).

## **Supplementary Table 7.** Ordered list of ROIs for the clustered correlation matrix of “CN to MCI” group (Figure 2B, second column).

| **Module #** | **Region #** | **Region Name** |
| --- | --- | --- |
| **Module 1** | 1 | ctx-lh-caudalanteriorcingulate |
|  | 2 | ctx-lh-caudalmiddlefrontal |
|  | 3 | ctx-lh-isthmuscingulate |
|  | 4 | ctx-lh-paracentral |
|  | 5 | ctx-lh-postcentral |
|  | 6 | ctx-lh-posteriorcingulate |
|  | 7 | ctx-lh-precentral |
|  | 8 | ctx-lh-precuneus |
|  | 9 | ctx-lh-superiorfrontal |
|  | 10 | ctx-lh-superiorparietal |
|  | 11 | ctx-lh-transversetemporal |
|  | 12 | ctx-rh-caudalanteriorcingulate |
|  | 13 | ctx-rh-caudalmiddlefrontal |
|  | 14 | ctx-rh-isthmuscingulate |
|  | 15 | ctx-rh-paracentral |
|  | 16 | ctx-rh-postcentral |
|  | 17 | ctx-rh-posteriorcingulate |
|  | 18 | ctx-rh-precentral |
|  | 19 | ctx-rh-precuneus |
|  | 20 | ctx-rh-superiorfrontal |
|  | 21 | ctx-rh-superiorparietal |
|  | 22 | ctx-rh-supramarginal |
|  | 23 | ctx-rh-transversetemporal |
| **Module 2** | 24 | ctx-lh-bankssts |
|  | 25 | ctx-lh-cuneus |
|  | 26 | ctx-lh-fusiform |
|  | 27 | ctx-lh-inferiorparietal |
|  | 28 | ctx-lh-inferiortemporal |
|  | 29 | ctx-lh-lateraloccipital |
|  | 30 | ctx-lh-lingual |
|  | 31 | ctx-lh-middletemporal |
|  | 32 | ctx-lh-parsopercularis |
|  | 33 | ctx-lh-parsorbitalis |
|  | 34 | ctx-lh-parstriangularis |
|  | 35 | ctx-lh-pericalcarine |
|  | 36 | ctx-lh-rostralmiddlefrontal |
|  | 37 | ctx-lh-superiortemporal |
|  | 38 | ctx-lh-supramarginal |
|  | 39 | ctx-lh-frontalpole |
|  | 40 | ctx-rh-bankssts |
|  | 41 | ctx-rh-cuneus |
|  | 42 | ctx-rh-inferiorparietal |
|  | 43 | ctx-rh-inferiortemporal |
|  | 44 | ctx-rh-lateraloccipital |
|  | 45 | ctx-rh-lingual |
|  | 46 | ctx-rh-middletemporal |
|  | 47 | ctx-rh-parsopercularis |
|  | 48 | ctx-rh-parsorbitalis |
|  | 49 | ctx-rh-parstriangularis |
|  | 50 | ctx-rh-pericalcarine |
|  | 51 | ctx-rh-rostralmiddlefrontal |
|  | 52 | ctx-rh-superiortemporal |
|  | 53 | ctx-rh-frontalpole |
| **Module 3** | 54 | Left-Hippocampus |
|  | 55 | Left-Amygdala |
|  | 56 | Right-Hippocampus |
|  | 57 | Right-Amygdala |
|  | 58 | ctx-lh-entorhinal |
|  | 59 | ctx-lh-lateralorbitofrontal |
|  | 60 | ctx-lh-medialorbitofrontal |
|  | 61 | ctx-lh-parahippocampal |
|  | 62 | ctx-lh-rostralanteriorcingulate |
|  | 63 | ctx-lh-temporalpole |
|  | 64 | ctx-lh-insula |
|  | 65 | ctx-rh-entorhinal |
|  | 66 | ctx-rh-fusiform |
|  | 67 | ctx-rh-lateralorbitofrontal |
|  | 68 | ctx-rh-medialorbitofrontal |
|  | 69 | ctx-rh-parahippocampal |
|  | 70 | ctx-rh-rostralanteriorcingulate |
|  | 71 | ctx-rh-temporalpole |
|  | 72 | ctx-rh-insula |

## **Supplementary Table 8.** Ordered list of ROIs for the clustered correlation matrix of “CN to MCI” group - women only (Supplementary Figure 2B, second column).

| **Module #** | **Region #** | **Region Name** |
| --- | --- | --- |
| **Module 1** | 1 | ctx-lh-inferiortemporal |
|  | 2 | ctx-lh-lateralorbitofrontal |
|  | 3 | ctx-lh-parsorbitalis |
|  | 4 | ctx-lh-frontalpole |
|  | 5 | ctx-rh-inferiortemporal |
|  | 6 | ctx-rh-lateralorbitofrontal |
|  | 7 | ctx-rh-parsopercularis |
|  | 8 | ctx-rh-parsorbitalis |
|  | 9 | ctx-rh-parstriangularis |
|  | 10 | ctx-rh-rostralmiddlefrontal |
|  | 11 | ctx-rh-frontalpole |
| **Module 2** | 12 | ctx-lh-bankssts |
|  | 13 | ctx-lh-caudalmiddlefrontal |
|  | 14 | ctx-lh-inferiorparietal |
|  | 15 | ctx-lh-isthmuscingulate |
|  | 16 | ctx-lh-lateraloccipital |
|  | 17 | ctx-lh-lingual |
|  | 18 | ctx-lh-middletemporal |
|  | 19 | ctx-lh-parsopercularis |
|  | 20 | ctx-lh-parstriangularis |
|  | 21 | ctx-lh-postcentral |
|  | 22 | ctx-lh-posteriorcingulate |
|  | 23 | ctx-lh-precentral |
|  | 24 | ctx-lh-precuneus |
|  | 25 | ctx-lh-rostralmiddlefrontal |
|  | 26 | ctx-lh-superiortemporal |
|  | 27 | ctx-lh-supramarginal |
|  | 28 | ctx-lh-transversetemporal |
|  | 29 | ctx-lh-insula |
|  | 30 | ctx-rh-bankssts |
|  | 31 | ctx-rh-caudalmiddlefrontal |
|  | 32 | ctx-rh-inferiorparietal |
|  | 33 | ctx-rh-isthmuscingulate |
|  | 34 | ctx-rh-lateraloccipital |
|  | 35 | ctx-rh-middletemporal |
|  | 36 | ctx-rh-postcentral |
|  | 37 | ctx-rh-posteriorcingulate |
|  | 38 | ctx-rh-precentral |
|  | 39 | ctx-rh-precuneus |
|  | 40 | ctx-rh-superiorparietal |
|  | 41 | ctx-rh-superiortemporal |
|  | 42 | ctx-rh-supramarginal |
|  | 43 | ctx-rh-transversetemporal |
|  | 44 | ctx-rh-insula |
| **Module 3** | 45 | Left-Hippocampus |
|  | 46 | Left-Amygdala |
|  | 47 | Right-Hippocampus |
|  | 48 | Right-Amygdala |
|  | 49 | ctx-lh-caudalanteriorcingulate |
|  | 50 | ctx-lh-cuneus |
|  | 51 | ctx-lh-entorhinal |
|  | 52 | ctx-lh-fusiform |
|  | 53 | ctx-lh-medialorbitofrontal |
|  | 54 | ctx-lh-parahippocampal |
|  | 55 | ctx-lh-paracentral |
|  | 56 | ctx-lh-pericalcarine |
|  | 57 | ctx-lh-rostralanteriorcingulate |
|  | 58 | ctx-lh-superiorfrontal |
|  | 59 | ctx-lh-superiorparietal |
|  | 60 | ctx-lh-temporalpole |
|  | 61 | ctx-rh-caudalanteriorcingulate |
|  | 62 | ctx-rh-cuneus |
|  | 63 | ctx-rh-entorhinal |
|  | 64 | ctx-rh-fusiform |
|  | 65 | ctx-rh-lingual |
|  | 66 | ctx-rh-medialorbitofrontal |
|  | 67 | ctx-rh-parahippocampal |
|  | 68 | ctx-rh-paracentral |
|  | 69 | ctx-rh-pericalcarine |
|  | 70 | ctx-rh-rostralanteriorcingulate |
|  | 71 | ctx-rh-superiorfrontal |
|  | 72 | ctx-rh-temporalpole |

## **Supplementary Table 9.** Ordered list of ROIs for the clustered correlation matrix of “CN to MCI” group - men only (Supplementary Figure 2E, second column).

| **Module #** | **Region #** | **Region Name** |
| --- | --- | --- |
| **Module 1** | 1 | ctx-lh-bankssts |
|  | 2 | ctx-lh-cuneus |
|  | 3 | ctx-lh-fusiform |
|  | 4 | ctx-lh-inferiorparietal |
|  | 5 | ctx-lh-inferiortemporal |
|  | 6 | ctx-lh-lateraloccipital |
|  | 7 | ctx-lh-lateralorbitofrontal |
|  | 8 | ctx-lh-lingual |
|  | 9 | ctx-lh-middletemporal |
|  | 10 | ctx-lh-parsopercularis |
|  | 11 | ctx-lh-parsorbitalis |
|  | 12 | ctx-lh-parstriangularis |
|  | 13 | ctx-lh-pericalcarine |
|  | 14 | ctx-lh-postcentral |
|  | 15 | ctx-lh-rostralmiddlefrontal |
|  | 16 | ctx-lh-superiorparietal |
|  | 17 | ctx-lh-superiortemporal |
|  | 18 | ctx-lh-supramarginal |
|  | 19 | ctx-lh-frontalpole |
|  | 20 | ctx-rh-bankssts |
|  | 21 | ctx-rh-cuneus |
|  | 22 | ctx-rh-fusiform |
|  | 23 | ctx-rh-inferiorparietal |
|  | 24 | ctx-rh-inferiortemporal |
|  | 25 | ctx-rh-lateraloccipital |
|  | 26 | ctx-rh-lateralorbitofrontal |
|  | 27 | ctx-rh-lingual |
|  | 28 | ctx-rh-middletemporal |
|  | 29 | ctx-rh-parsopercularis |
|  | 30 | ctx-rh-parsorbitalis |
|  | 31 | ctx-rh-parstriangularis |
|  | 32 | ctx-rh-pericalcarine |
|  | 33 | ctx-rh-rostralmiddlefrontal |
|  | 34 | ctx-rh-superiorparietal |
|  | 35 | ctx-rh-superiortemporal |
|  | 36 | ctx-rh-supramarginal |
|  | 37 | ctx-rh-frontalpole |
| **Module 2** | 38 | Left-Hippocampus |
|  | 39 | Left-Amygdala |
|  | 40 | Right-Hippocampus |
|  | 41 | Right-Amygdala |
|  | 42 | ctx-lh-caudalanteriorcingulate |
|  | 43 | ctx-lh-caudalmiddlefrontal |
|  | 44 | ctx-lh-entorhinal |
|  | 45 | ctx-lh-isthmuscingulate |
|  | 46 | ctx-lh-medialorbitofrontal |
|  | 47 | ctx-lh-parahippocampal |
|  | 48 | ctx-lh-paracentral |
|  | 49 | ctx-lh-posteriorcingulate |
|  | 50 | ctx-lh-precentral |
|  | 51 | ctx-lh-precuneus |
|  | 52 | ctx-lh-rostralanteriorcingulate |
|  | 53 | ctx-lh-superiorfrontal |
|  | 54 | ctx-lh-temporalpole |
|  | 55 | ctx-lh-transversetemporal |
|  | 56 | ctx-lh-insula |
|  | 57 | ctx-rh-caudalanteriorcingulate |
|  | 58 | ctx-rh-caudalmiddlefrontal |
|  | 59 | ctx-rh-entorhinal |
|  | 60 | ctx-rh-isthmuscingulate |
|  | 61 | ctx-rh-medialorbitofrontal |
|  | 62 | ctx-rh-parahippocampal |
|  | 63 | ctx-rh-paracentral |
|  | 64 | ctx-rh-postcentral |
|  | 65 | ctx-rh-posteriorcingulate |
|  | 66 | ctx-rh-precentral |
|  | 67 | ctx-rh-precuneus |
|  | 68 | ctx-rh-rostralanteriorcingulate |
|  | 69 | ctx-rh-superiorfrontal |
|  | 70 | ctx-rh-temporalpole |
|  | 71 | ctx-rh-transversetemporal |
|  | 72 | ctx-rh-insula |

### CN to AD

Table A8 shows the ordered list of ROIs for the clustered correlation matrix of “CN to AD” group (Figure 2B, third column). Table A9 shows the ordered list of ROIs for the clustered correlation matrix of women in “CN to AD” group (Supplementary Figure 2B, third column). Table A10 shows the ordered list of ROIs for the clustered correlation matrix of men in “CN to AD” group (Supplementary Figure 2E, third column).

## **Supplementary Table 10.** Ordered list of ROIs for the clustered correlation matrix of “CN to AD” group (Figure 2B, third column).

| **Module #** | **Region #** | **Region Name** |
| --- | --- | --- |
| **Module 1** | 1 | ctx-lh-bankssts |
|  | 2 | ctx-lh-caudalanteriorcingulate |
|  | 3 | ctx-lh-caudalmiddlefrontal |
|  | 4 | ctx-lh-inferiorparietal |
|  | 5 | ctx-lh-isthmuscingulate |
|  | 6 | ctx-lh-medialorbitofrontal |
|  | 7 | ctx-lh-paracentral |
|  | 8 | ctx-lh-parsopercularis |
|  | 9 | ctx-lh-parstriangularis |
|  | 10 | ctx-lh-postcentral |
|  | 11 | ctx-lh-posteriorcingulate |
|  | 12 | ctx-lh-precentral |
|  | 13 | ctx-lh-precuneus |
|  | 14 | ctx-lh-rostralmiddlefrontal |
|  | 15 | ctx-lh-superiorfrontal |
|  | 16 | ctx-lh-superiorparietal |
|  | 17 | ctx-lh-supramarginal |
|  | 18 | ctx-lh-frontalpole |
|  | 19 | ctx-lh-insula |
|  | 20 | ctx-rh-caudalanteriorcingulate |
|  | 21 | ctx-rh-caudalmiddlefrontal |
|  | 22 | ctx-rh-inferiorparietal |
|  | 23 | ctx-rh-isthmuscingulate |
|  | 24 | ctx-rh-paracentral |
|  | 25 | ctx-rh-parstriangularis |
|  | 26 | ctx-rh-postcentral |
|  | 27 | ctx-rh-posteriorcingulate |
|  | 28 | ctx-rh-precentral |
|  | 29 | ctx-rh-precuneus |
|  | 30 | ctx-rh-rostralanteriorcingulate |
|  | 31 | ctx-rh-rostralmiddlefrontal |
|  | 32 | ctx-rh-superiorfrontal |
|  | 33 | ctx-rh-superiorparietal |
|  | 34 | ctx-rh-supramarginal |
|  | 35 | ctx-rh-frontalpole |
| **Module 2** | 36 | ctx-lh-cuneus |
|  | 37 | ctx-lh-lingual |
|  | 38 | ctx-lh-pericalcarine |
|  | 39 | ctx-lh-rostralanteriorcingulate |
|  | 40 | ctx-rh-bankssts |
|  | 41 | ctx-rh-lingual |
|  | 42 | ctx-rh-parsopercularis |
|  | 43 | ctx-rh-pericalcarine |
|  | 44 | ctx-rh-transversetemporal |
| **Module 3** | 45 | Left-Hippocampus |
|  | 46 | Left-Amygdala |
|  | 47 | Right-Hippocampus |
|  | 48 | Right-Amygdala |
|  | 49 | ctx-lh-entorhinal |
|  | 50 | ctx-lh-fusiform |
|  | 51 | ctx-lh-inferiortemporal |
|  | 52 | ctx-lh-lateraloccipital |
|  | 53 | ctx-lh-lateralorbitofrontal |
|  | 54 | ctx-lh-middletemporal |
|  | 55 | ctx-lh-parahippocampal |
|  | 56 | ctx-lh-parsorbitalis |
|  | 57 | ctx-lh-superiortemporal |
|  | 58 | ctx-lh-temporalpole |
|  | 59 | ctx-lh-transversetemporal |
|  | 60 | ctx-rh-cuneus |
|  | 61 | ctx-rh-entorhinal |
|  | 62 | ctx-rh-fusiform |
|  | 63 | ctx-rh-inferiortemporal |
|  | 64 | ctx-rh-lateraloccipital |
|  | 65 | ctx-rh-lateralorbitofrontal |
|  | 66 | ctx-rh-medialorbitofrontal |
|  | 67 | ctx-rh-middletemporal |
|  | 68 | ctx-rh-parahippocampal |
|  | 69 | ctx-rh-parsorbitalis |
|  | 70 | ctx-rh-superiortemporal |
|  | 71 | ctx-rh-temporalpole |
|  | 72 | ctx-rh-insula |

## **Supplementary Table 11.** Ordered list of ROIs for the clustered correlation matrix of “CN to AD” group - women only (Supplementary Figure 2B, third column).

| **Module #** | **Region #** | **Region Name** |
| --- | --- | --- |
| **Module 1** | 1 | ctx-lh-caudalanteriorcingulate |
|  | 2 | ctx-lh-lateralorbitofrontal |
|  | 3 | ctx-lh-medialorbitofrontal |
|  | 4 | ctx-lh-parsorbitalis |
|  | 5 | ctx-lh-posteriorcingulate |
|  | 6 | ctx-lh-rostralmiddlefrontal |
|  | 7 | ctx-lh-frontalpole |
|  | 8 | ctx-rh-caudalanteriorcingulate |
|  | 9 | ctx-rh-cuneus |
|  | 10 | ctx-rh-inferiorparietal |
|  | 11 | ctx-rh-lateraloccipital |
|  | 12 | ctx-rh-lateralorbitofrontal |
|  | 13 | ctx-rh-medialorbitofrontal |
|  | 14 | ctx-rh-parsorbitalis |
|  | 15 | ctx-rh-parstriangularis |
|  | 16 | ctx-rh-posteriorcingulate |
|  | 17 | ctx-rh-rostralmiddlefrontal |
|  | 18 | ctx-rh-superiortemporal |
|  | 19 | ctx-rh-frontalpole |
| **Module 2** | 20 | ctx-lh-caudalmiddlefrontal |
|  | 21 | ctx-lh-cuneus |
|  | 22 | ctx-lh-inferiorparietal |
|  | 23 | ctx-lh-isthmuscingulate |
|  | 24 | ctx-lh-lingual |
|  | 25 | ctx-lh-paracentral |
|  | 26 | ctx-lh-parsopercularis |
|  | 27 | ctx-lh-parstriangularis |
|  | 28 | ctx-lh-pericalcarine |
|  | 29 | ctx-lh-postcentral |
|  | 30 | ctx-lh-precentral |
|  | 31 | ctx-lh-precuneus |
|  | 32 | ctx-lh-rostralanteriorcingulate |
|  | 33 | ctx-lh-superiorfrontal |
|  | 34 | ctx-lh-superiorparietal |
|  | 35 | ctx-lh-supramarginal |
|  | 36 | ctx-lh-insula |
|  | 37 | ctx-rh-bankssts |
|  | 38 | ctx-rh-caudalmiddlefrontal |
|  | 39 | ctx-rh-isthmuscingulate |
|  | 40 | ctx-rh-lingual |
|  | 41 | ctx-rh-paracentral |
|  | 42 | ctx-rh-parsopercularis |
|  | 43 | ctx-rh-pericalcarine |
|  | 44 | ctx-rh-postcentral |
|  | 45 | ctx-rh-precentral |
|  | 46 | ctx-rh-precuneus |
|  | 47 | ctx-rh-rostralanteriorcingulate |
|  | 48 | ctx-rh-superiorfrontal |
|  | 49 | ctx-rh-superiorparietal |
|  | 50 | ctx-rh-supramarginal |
|  | 51 | ctx-rh-transversetemporal |
|  | 52 | ctx-rh-insula |
| **Module 3** | 53 | Left-Hippocampus |
|  | 54 | Left-Amygdala |
|  | 55 | Right-Hippocampus |
|  | 56 | Right-Amygdala |
|  | 57 | ctx-lh-bankssts |
|  | 58 | ctx-lh-entorhinal |
|  | 59 | ctx-lh-fusiform |
|  | 60 | ctx-lh-inferiortemporal |
|  | 61 | ctx-lh-lateraloccipital |
|  | 62 | ctx-lh-middletemporal |
|  | 63 | ctx-lh-parahippocampal |
|  | 64 | ctx-lh-superiortemporal |
|  | 65 | ctx-lh-temporalpole |
|  | 66 | ctx-lh-transversetemporal |
|  | 67 | ctx-rh-entorhinal |
|  | 68 | ctx-rh-fusiform |
|  | 69 | ctx-rh-inferiortemporal |
|  | 70 | ctx-rh-middletemporal |
|  | 71 | ctx-rh-parahippocampal |
|  | 72 | ctx-rh-temporalpole |

## **Supplementary Table 12.** Ordered list of ROIs for the clustered correlation matrix of “CN to AD” group - men only (Supplementary Figure 2E, third column).

| **Module #** | **Region #** | **Region Name** |
| --- | --- | --- |
| **Module 1** | 1 | ctx-lh-caudalmiddlefrontal |
|  | 2 | ctx-lh-cuneus |
|  | 3 | ctx-lh-inferiorparietal |
|  | 4 | ctx-lh-lateraloccipital |
|  | 5 | ctx-lh-lingual |
|  | 6 | ctx-lh-paracentral |
|  | 7 | ctx-lh-parsopercularis |
|  | 8 | ctx-lh-pericalcarine |
|  | 9 | ctx-lh-postcentral |
|  | 10 | ctx-lh-precentral |
|  | 11 | ctx-lh-rostralmiddlefrontal |
|  | 12 | ctx-lh-superiorparietal |
|  | 13 | ctx-lh-supramarginal |
|  | 14 | ctx-lh-temporalpole |
|  | 15 | ctx-lh-transversetemporal |
|  | 16 | ctx-rh-bankssts |
|  | 17 | ctx-rh-caudalmiddlefrontal |
|  | 18 | ctx-rh-cuneus |
|  | 19 | ctx-rh-entorhinal |
|  | 20 | ctx-rh-inferiorparietal |
|  | 21 | ctx-rh-inferiortemporal |
|  | 22 | ctx-rh-lateraloccipital |
|  | 23 | ctx-rh-lingual |
|  | 24 | ctx-rh-middletemporal |
|  | 25 | ctx-rh-parahippocampal |
|  | 26 | ctx-rh-paracentral |
|  | 27 | ctx-rh-parstriangularis |
|  | 28 | ctx-rh-pericalcarine |
|  | 29 | ctx-rh-postcentral |
|  | 30 | ctx-rh-precentral |
|  | 31 | ctx-rh-rostralanteriorcingulate |
|  | 32 | ctx-rh-rostralmiddlefrontal |
|  | 33 | ctx-rh-superiorfrontal |
|  | 34 | ctx-rh-superiorparietal |
|  | 35 | ctx-rh-superiortemporal |
|  | 36 | ctx-rh-supramarginal |
|  | 37 | ctx-rh-temporalpole |
|  | 38 | ctx-rh-transversetemporal |
| **Module 2** | 39 | Left-Hippocampus |
|  | 40 | Left-Amygdala |
|  | 41 | Right-Hippocampus |
|  | 42 | Right-Amygdala |
|  | 43 | ctx-lh-bankssts |
|  | 44 | ctx-lh-caudalanteriorcingulate |
|  | 45 | ctx-lh-entorhinal |
|  | 46 | ctx-lh-fusiform |
|  | 47 | ctx-lh-inferiortemporal |
|  | 48 | ctx-lh-isthmuscingulate |
|  | 49 | ctx-lh-lateralorbitofrontal |
|  | 50 | ctx-lh-medialorbitofrontal |
|  | 51 | ctx-lh-middletemporal |
|  | 52 | ctx-lh-parahippocampal |
|  | 53 | ctx-lh-parsorbitalis |
|  | 54 | ctx-lh-parstriangularis |
|  | 55 | ctx-lh-posteriorcingulate |
|  | 56 | ctx-lh-precuneus |
|  | 57 | ctx-lh-rostralanteriorcingulate |
|  | 58 | ctx-lh-superiorfrontal |
|  | 59 | ctx-lh-superiortemporal |
|  | 60 | ctx-lh-frontalpole |
|  | 61 | ctx-lh-insula |
|  | 62 | ctx-rh-caudalanteriorcingulate |
|  | 63 | ctx-rh-fusiform |
|  | 64 | ctx-rh-isthmuscingulate |
|  | 65 | ctx-rh-lateralorbitofrontal |
|  | 66 | ctx-rh-medialorbitofrontal |
|  | 67 | ctx-rh-parsopercularis |
|  | 68 | ctx-rh-parsorbitalis |
|  | 69 | ctx-rh-posteriorcingulate |
|  | 70 | ctx-rh-precuneus |
|  | 71 | ctx-rh-frontalpole |
|  | 72 | ctx-rh-insula |

### AD

Table A11 shows the ordered list of ROIs for the clustered correlation matrix of “AD” group (Figure 2B, fourth column). Table A12 shows the ordered list of ROIs for the clustered correlation matrix of women in “AD” group (Supplementary Figure 2B, fourth column). Table A13 shows the ordered list of ROIs for the clustered correlation matrix of men in “AD” group (Supplementary Figure 2E, fourth column).

## **Supplementary Table 13.** Ordered list of ROIs for the clustered correlation matrix of “AD” group (Figure 2B, fourth column).

| **Module #** | **Region #** | **Region Name** |
| --- | --- | --- |
| **Module 1** | 1 | ctx-lh-caudalmiddlefrontal |
|  | 2 | ctx-lh-parsopercularis |
|  | 3 | ctx-lh-parsorbitalis |
|  | 4 | ctx-lh-parstriangularis |
|  | 5 | ctx-lh-rostralmiddlefrontal |
|  | 6 | ctx-lh-superiorfrontal |
|  | 7 | ctx-lh-frontalpole |
|  | 8 | ctx-rh-caudalmiddlefrontal |
|  | 9 | ctx-rh-parsopercularis |
|  | 10 | ctx-rh-parsorbitalis |
|  | 11 | ctx-rh-parstriangularis |
|  | 12 | ctx-rh-rostralmiddlefrontal |
|  | 13 | ctx-rh-superiorfrontal |
|  | 14 | ctx-rh-frontalpole |
| **Module 2** | 15 | ctx-lh-bankssts |
|  | 16 | ctx-lh-cuneus |
|  | 17 | ctx-lh-fusiform |
|  | 18 | ctx-lh-inferiorparietal |
|  | 19 | ctx-lh-inferiortemporal |
|  | 20 | ctx-lh-lateraloccipital |
|  | 21 | ctx-lh-lingual |
|  | 22 | ctx-lh-middletemporal |
|  | 23 | ctx-lh-paracentral |
|  | 24 | ctx-lh-pericalcarine |
|  | 25 | ctx-lh-postcentral |
|  | 26 | ctx-lh-precentral |
|  | 27 | ctx-lh-precuneus |
|  | 28 | ctx-lh-superiorparietal |
|  | 29 | ctx-lh-superiortemporal |
|  | 30 | ctx-lh-supramarginal |
|  | 31 | ctx-lh-transversetemporal |
|  | 32 | ctx-rh-bankssts |
|  | 33 | ctx-rh-cuneus |
|  | 34 | ctx-rh-fusiform |
|  | 35 | ctx-rh-inferiorparietal |
|  | 36 | ctx-rh-inferiortemporal |
|  | 37 | ctx-rh-lateraloccipital |
|  | 38 | ctx-rh-lingual |
|  | 39 | ctx-rh-middletemporal |
|  | 40 | ctx-rh-paracentral |
|  | 41 | ctx-rh-pericalcarine |
|  | 42 | ctx-rh-postcentral |
|  | 43 | ctx-rh-precentral |
|  | 44 | ctx-rh-precuneus |
|  | 45 | ctx-rh-superiorparietal |
|  | 46 | ctx-rh-superiortemporal |
|  | 47 | ctx-rh-supramarginal |
|  | 48 | ctx-rh-transversetemporal |
| **Module 3** | 49 | Left-Hippocampus |
|  | 50 | Left-Amygdala |
|  | 51 | Right-Hippocampus |
|  | 52 | Right-Amygdala |
|  | 53 | ctx-lh-caudalanteriorcingulate |
|  | 54 | ctx-lh-entorhinal |
|  | 55 | ctx-lh-isthmuscingulate |
|  | 56 | ctx-lh-lateralorbitofrontal |
|  | 57 | ctx-lh-medialorbitofrontal |
|  | 58 | ctx-lh-parahippocampal |
|  | 59 | ctx-lh-posteriorcingulate |
|  | 60 | ctx-lh-rostralanteriorcingulate |
|  | 61 | ctx-lh-temporalpole |
|  | 62 | ctx-lh-insula |
|  | 63 | ctx-rh-caudalanteriorcingulate |
|  | 64 | ctx-rh-entorhinal |
|  | 65 | ctx-rh-isthmuscingulate |
|  | 66 | ctx-rh-lateralorbitofrontal |
|  | 67 | ctx-rh-medialorbitofrontal |
|  | 68 | ctx-rh-parahippocampal |
|  | 69 | ctx-rh-posteriorcingulate |
|  | 70 | ctx-rh-rostralanteriorcingulate |
|  | 71 | ctx-rh-temporalpole |
|  | 72 | ctx-rh-insula |

## **Supplementary Table 14.** Ordered list of ROIs for the clustered correlation matrix of “AD” group - women only (Supplementary Figure 2B, fourth column).

| **Module #** | **Region #** | **Region Name** |
| --- | --- | --- |
| **Module 1** | 1 | ctx-lh-caudalmiddlefrontal |
|  | 2 | ctx-lh-parsopercularis |
|  | 3 | ctx-lh-parsorbitalis |
|  | 4 | ctx-lh-parstriangularis |
|  | 5 | ctx-lh-rostralmiddlefrontal |
|  | 6 | ctx-lh-superiorfrontal |
|  | 7 | ctx-lh-frontalpole |
|  | 8 | ctx-rh-caudalmiddlefrontal |
|  | 9 | ctx-rh-parsopercularis |
|  | 10 | ctx-rh-parsorbitalis |
|  | 11 | ctx-rh-parstriangularis |
|  | 12 | ctx-rh-rostralmiddlefrontal |
|  | 13 | ctx-rh-superiorfrontal |
|  | 14 | ctx-rh-frontalpole |
| **Module 2** | 15 | ctx-lh-bankssts |
|  | 16 | ctx-lh-cuneus |
|  | 17 | ctx-lh-fusiform |
|  | 18 | ctx-lh-inferiorparietal |
|  | 19 | ctx-lh-inferiortemporal |
|  | 20 | ctx-lh-lateraloccipital |
|  | 21 | ctx-lh-lingual |
|  | 22 | ctx-lh-middletemporal |
|  | 23 | ctx-lh-paracentral |
|  | 24 | ctx-lh-pericalcarine |
|  | 25 | ctx-lh-postcentral |
|  | 26 | ctx-lh-precentral |
|  | 27 | ctx-lh-precuneus |
|  | 28 | ctx-lh-superiorparietal |
|  | 29 | ctx-lh-superiortemporal |
|  | 30 | ctx-lh-supramarginal |
|  | 31 | ctx-lh-transversetemporal |
|  | 32 | ctx-rh-bankssts |
|  | 33 | ctx-rh-cuneus |
|  | 34 | ctx-rh-fusiform |
|  | 35 | ctx-rh-inferiorparietal |
|  | 36 | ctx-rh-inferiortemporal |
|  | 37 | ctx-rh-lateraloccipital |
|  | 38 | ctx-rh-lingual |
|  | 39 | ctx-rh-middletemporal |
|  | 40 | ctx-rh-paracentral |
|  | 41 | ctx-rh-pericalcarine |
|  | 42 | ctx-rh-postcentral |
|  | 43 | ctx-rh-precentral |
|  | 44 | ctx-rh-precuneus |
|  | 45 | ctx-rh-superiorparietal |
|  | 46 | ctx-rh-superiortemporal |
|  | 47 | ctx-rh-supramarginal |
|  | 48 | ctx-rh-transversetemporal |
| **Module 3** | 49 | Left-Hippocampus |
|  | 50 | Left-Amygdala |
|  | 51 | Right-Hippocampus |
|  | 52 | Right-Amygdala |
|  | 53 | ctx-lh-caudalanteriorcingulate |
|  | 54 | ctx-lh-entorhinal |
|  | 55 | ctx-lh-isthmuscingulate |
|  | 56 | ctx-lh-lateralorbitofrontal |
|  | 57 | ctx-lh-medialorbitofrontal |
|  | 58 | ctx-lh-parahippocampal |
|  | 59 | ctx-lh-posteriorcingulate |
|  | 60 | ctx-lh-rostralanteriorcingulate |
|  | 61 | ctx-lh-temporalpole |
|  | 62 | ctx-lh-insula |
|  | 63 | ctx-rh-caudalanteriorcingulate |
|  | 64 | ctx-rh-entorhinal |
|  | 65 | ctx-rh-isthmuscingulate |
|  | 66 | ctx-rh-lateralorbitofrontal |
|  | 67 | ctx-rh-medialorbitofrontal |
|  | 68 | ctx-rh-parahippocampal |
|  | 69 | ctx-rh-posteriorcingulate |
|  | 70 | ctx-rh-rostralanteriorcingulate |
|  | 71 | ctx-rh-temporalpole |
|  | 72 | ctx-rh-insula |

## **Supplementary Table 15.** Ordered list of ROIs for the clustered correlation matrix of “AD” group - men only (Supplementary Figure 2E, fourth column).

| **Module #** | **Region #** | **Region Name** |
| --- | --- | --- |
| **Module 1** | 1 | ctx-lh-caudalmiddlefrontal |
|  | 2 | ctx-lh-paracentral |
|  | 3 | ctx-lh-parsopercularis |
|  | 4 | ctx-lh-parsorbitalis |
|  | 5 | ctx-lh-parstriangularis |
|  | 6 | ctx-lh-precentral |
|  | 7 | ctx-lh-rostralmiddlefrontal |
|  | 8 | ctx-lh-superiorfrontal |
|  | 9 | ctx-lh-frontalpole |
|  | 10 | ctx-rh-caudalmiddlefrontal |
|  | 11 | ctx-rh-paracentral |
|  | 12 | ctx-rh-parsopercularis |
|  | 13 | ctx-rh-parsorbitalis |
|  | 14 | ctx-rh-parstriangularis |
|  | 15 | ctx-rh-precentral |
|  | 16 | ctx-rh-rostralmiddlefrontal |
|  | 17 | ctx-rh-superiorfrontal |
|  | 18 | ctx-rh-frontalpole |
| **Module 2** | 19 | ctx-lh-bankssts |
|  | 20 | ctx-lh-cuneus |
|  | 21 | ctx-lh-fusiform |
|  | 22 | ctx-lh-inferiorparietal |
|  | 23 | ctx-lh-inferiortemporal |
|  | 24 | ctx-lh-lateraloccipital |
|  | 25 | ctx-lh-lingual |
|  | 26 | ctx-lh-middletemporal |
|  | 27 | ctx-lh-pericalcarine |
|  | 28 | ctx-lh-postcentral |
|  | 29 | ctx-lh-precuneus |
|  | 30 | ctx-lh-superiorparietal |
|  | 31 | ctx-lh-superiortemporal |
|  | 32 | ctx-lh-supramarginal |
|  | 33 | ctx-lh-transversetemporal |
|  | 34 | ctx-rh-bankssts |
|  | 35 | ctx-rh-cuneus |
|  | 36 | ctx-rh-fusiform |
|  | 37 | ctx-rh-inferiorparietal |
|  | 38 | ctx-rh-inferiortemporal |
|  | 39 | ctx-rh-isthmuscingulate |
|  | 40 | ctx-rh-lateraloccipital |
|  | 41 | ctx-rh-lingual |
|  | 42 | ctx-rh-middletemporal |
|  | 43 | ctx-rh-pericalcarine |
|  | 44 | ctx-rh-postcentral |
|  | 45 | ctx-rh-precuneus |
|  | 46 | ctx-rh-superiorparietal |
|  | 47 | ctx-rh-superiortemporal |
|  | 48 | ctx-rh-supramarginal |
|  | 49 | ctx-rh-transversetemporal |
| **Module 3** | 50 | Left-Hippocampus |
|  | 51 | Left-Amygdala |
|  | 52 | Right-Hippocampus |
|  | 53 | Right-Amygdala |
|  | 54 | ctx-lh-caudalanteriorcingulate |
|  | 55 | ctx-lh-entorhinal |
|  | 56 | ctx-lh-isthmuscingulate |
|  | 57 | ctx-lh-lateralorbitofrontal |
|  | 58 | ctx-lh-medialorbitofrontal |
|  | 59 | ctx-lh-parahippocampal |
|  | 60 | ctx-lh-posteriorcingulate |
|  | 61 | ctx-lh-rostralanteriorcingulate |
|  | 62 | ctx-lh-temporalpole |
|  | 63 | ctx-lh-insula |
|  | 64 | ctx-rh-caudalanteriorcingulate |
|  | 65 | ctx-rh-entorhinal |
|  | 66 | ctx-rh-lateralorbitofrontal |
|  | 67 | ctx-rh-medialorbitofrontal |
|  | 68 | ctx-rh-parahippocampal |
|  | 69 | ctx-rh-posteriorcingulate |
|  | 70 | ctx-rh-rostralanteriorcingulate |
|  | 71 | ctx-rh-temporalpole |
|  | 72 | ctx-rh-insula |

# Appendix B: Mean and Standard Deviation of FDG SUVR

### CN to CN

## **Supplementary Table 16.** Mean and standard deviation of FDG SUVR within the “CN to CN” group.

|  | **All**  **mean** | **All**  **std** | **Women**  **mean** | **Women**  **std** | **Men**  **mean** | **Men**  **std** |
| --- | --- | --- | --- | --- | --- | --- |
| **ctx-lh-bankssts** | 1.586 | 0.18 | 1.586 | 0.131 | 1.586 | 0.211 |
| **ctx-lh-caudalanteriorcingulate** | 1.345 | 0.163 | 1.342 | 0.143 | 1.347 | 0.178 |
| **ctx-lh-caudalmiddlefrontal** | 1.609 | 0.208 | 1.598 | 0.134 | 1.617 | 0.251 |
| **ctx-lh-cuneus** | 1.742 | 0.233 | 1.693 | 0.179 | 1.779 | 0.262 |
| **ctx-lh-entorhinal** | 1.054 | 0.083 | 1.064 | 0.074 | 1.047 | 0.09 |
| **ctx-lh-fusiform** | 1.398 | 0.129 | 1.394 | 0.094 | 1.402 | 0.152 |
| **ctx-lh-inferiorparietal** | 1.497 | 0.203 | 1.499 | 0.139 | 1.496 | 0.242 |
| **ctx-lh-inferiortemporal** | 1.359 | 0.126 | 1.354 | 0.111 | 1.363 | 0.138 |
| **ctx-lh-isthmuscingulate** | 1.619 | 0.171 | 1.632 | 0.132 | 1.61 | 0.196 |
| **ctx-lh-lateraloccipital** | 1.475 | 0.203 | 1.46 | 0.154 | 1.487 | 0.235 |
| **ctx-lh-lateralorbitofrontal** | 1.485 | 0.145 | 1.456 | 0.105 | 1.507 | 0.167 |
| **ctx-lh-lingual** | 1.637 | 0.179 | 1.634 | 0.142 | 1.64 | 0.205 |
| **ctx-lh-medialorbitofrontal** | 1.447 | 0.142 | 1.422 | 0.098 | 1.466 | 0.166 |
| **ctx-lh-middletemporal** | 1.363 | 0.157 | 1.363 | 0.129 | 1.363 | 0.177 |
| **ctx-lh-parahippocampal** | 1.176 | 0.101 | 1.186 | 0.084 | 1.167 | 0.112 |
| **ctx-lh-paracentral** | 1.484 | 0.176 | 1.477 | 0.131 | 1.489 | 0.205 |
| **ctx-lh-parsopercularis** | 1.582 | 0.206 | 1.557 | 0.125 | 1.601 | 0.25 |
| **ctx-lh-parsorbitalis** | 1.471 | 0.213 | 1.436 | 0.174 | 1.499 | 0.237 |
| **ctx-lh-parstriangularis** | 1.544 | 0.232 | 1.519 | 0.133 | 1.563 | 0.286 |
| **ctx-lh-pericalcarine** | 1.788 | 0.22 | 1.754 | 0.19 | 1.814 | 0.239 |
| **ctx-lh-postcentral** | 1.396 | 0.19 | 1.381 | 0.105 | 1.408 | 0.236 |
| **ctx-lh-posteriorcingulate** | 1.589 | 0.189 | 1.598 | 0.127 | 1.582 | 0.226 |
| **ctx-lh-precentral** | 1.486 | 0.184 | 1.469 | 0.113 | 1.498 | 0.224 |
| **ctx-lh-precuneus** | 1.736 | 0.2 | 1.724 | 0.144 | 1.745 | 0.235 |
| **ctx-lh-rostralanteriorcingulate** | 1.395 | 0.155 | 1.395 | 0.134 | 1.394 | 0.17 |
| **ctx-lh-rostralmiddlefrontal** | 1.583 | 0.209 | 1.575 | 0.128 | 1.589 | 0.256 |
| **ctx-lh-superiorfrontal** | 1.468 | 0.17 | 1.47 | 0.115 | 1.467 | 0.203 |
| **ctx-lh-superiorparietal** | 1.425 | 0.191 | 1.416 | 0.144 | 1.432 | 0.221 |
| **ctx-lh-superiortemporal** | 1.305 | 0.143 | 1.296 | 0.1 | 1.311 | 0.17 |
| **ctx-lh-supramarginal** | 1.455 | 0.192 | 1.449 | 0.121 | 1.459 | 0.234 |
| **ctx-lh-frontalpole** | 1.366 | 0.214 | 1.35 | 0.168 | 1.379 | 0.245 |
| **ctx-lh-temporalpole** | 1.015 | 0.094 | 1.016 | 0.081 | 1.013 | 0.103 |
| **ctx-lh-transversetemporal** | 1.729 | 0.233 | 1.709 | 0.156 | 1.744 | 0.279 |
| **ctx-lh-insula** | 1.345 | 0.128 | 1.336 | 0.091 | 1.352 | 0.151 |
| **ctx-rh-bankssts** | 1.588 | 0.163 | 1.584 | 0.117 | 1.591 | 0.192 |
| **ctx-rh-caudalanteriorcingulate** | 1.372 | 0.163 | 1.381 | 0.131 | 1.366 | 0.185 |
| **ctx-rh-caudalmiddlefrontal** | 1.603 | 0.207 | 1.588 | 0.111 | 1.615 | 0.257 |
| **ctx-rh-cuneus** | 1.709 | 0.234 | 1.686 | 0.188 | 1.727 | 0.264 |
| **ctx-rh-entorhinal** | 1.042 | 0.103 | 1.035 | 0.115 | 1.047 | 0.093 |
| **ctx-rh-fusiform** | 1.387 | 0.125 | 1.388 | 0.084 | 1.387 | 0.149 |
| **ctx-rh-inferiorparietal** | 1.506 | 0.207 | 1.499 | 0.135 | 1.511 | 0.25 |
| **ctx-rh-inferiortemporal** | 1.36 | 0.12 | 1.358 | 0.099 | 1.361 | 0.134 |
| **ctx-rh-isthmuscingulate** | 1.647 | 0.17 | 1.651 | 0.122 | 1.645 | 0.2 |
| **ctx-rh-lateraloccipital** | 1.49 | 0.203 | 1.47 | 0.152 | 1.506 | 0.236 |
| **ctx-rh-lateralorbitofrontal** | 1.476 | 0.141 | 1.451 | 0.106 | 1.495 | 0.162 |
| **ctx-rh-lingual** | 1.627 | 0.17 | 1.615 | 0.125 | 1.635 | 0.199 |
| **ctx-rh-medialorbitofrontal** | 1.465 | 0.147 | 1.447 | 0.1 | 1.479 | 0.174 |
| **ctx-rh-middletemporal** | 1.379 | 0.154 | 1.383 | 0.122 | 1.377 | 0.176 |
| **ctx-rh-parahippocampal** | 1.169 | 0.092 | 1.181 | 0.078 | 1.159 | 0.101 |
| **ctx-rh-paracentral** | 1.481 | 0.173 | 1.481 | 0.124 | 1.481 | 0.204 |
| **ctx-rh-parsopercularis** | 1.606 | 0.215 | 1.573 | 0.117 | 1.63 | 0.266 |
| **ctx-rh-parsorbitalis** | 1.492 | 0.204 | 1.464 | 0.172 | 1.514 | 0.225 |
| **ctx-rh-parstriangularis** | 1.551 | 0.227 | 1.519 | 0.122 | 1.576 | 0.281 |
| **ctx-rh-pericalcarine** | 1.833 | 0.232 | 1.801 | 0.186 | 1.857 | 0.261 |
| **ctx-rh-postcentral** | 1.407 | 0.187 | 1.389 | 0.11 | 1.421 | 0.23 |
| **ctx-rh-posteriorcingulate** | 1.596 | 0.189 | 1.616 | 0.125 | 1.581 | 0.226 |
| **ctx-rh-precentral** | 1.491 | 0.185 | 1.473 | 0.117 | 1.506 | 0.223 |
| **ctx-rh-precuneus** | 1.723 | 0.202 | 1.718 | 0.132 | 1.727 | 0.244 |
| **ctx-rh-rostralanteriorcingulate** | 1.383 | 0.153 | 1.38 | 0.152 | 1.385 | 0.156 |
| **ctx-rh-rostralmiddlefrontal** | 1.591 | 0.205 | 1.581 | 0.109 | 1.599 | 0.256 |
| **ctx-rh-superiorfrontal** | 1.471 | 0.166 | 1.473 | 0.11 | 1.47 | 0.199 |
| **ctx-rh-superiorparietal** | 1.41 | 0.191 | 1.402 | 0.141 | 1.415 | 0.222 |
| **ctx-rh-superiortemporal** | 1.327 | 0.154 | 1.324 | 0.089 | 1.33 | 0.19 |
| **ctx-rh-supramarginal** | 1.448 | 0.199 | 1.436 | 0.107 | 1.457 | 0.248 |
| **ctx-rh-frontalpole** | 1.396 | 0.213 | 1.367 | 0.178 | 1.418 | 0.236 |
| **ctx-rh-temporalpole** | 1.001 | 0.099 | 1.008 | 0.102 | 0.997 | 0.097 |
| **ctx-rh-transversetemporal** | 1.703 | 0.235 | 1.687 | 0.157 | 1.716 | 0.281 |
| **ctx-rh-insula** | 1.342 | 0.134 | 1.336 | 0.089 | 1.348 | 0.161 |

### CN to MCI

## **Supplementary Table 17.** Mean and standard deviation of FDG SUVR within the “CN to MCI” group.

|  | **All**  **mean** | **All**  **std** | **Women**  **mean** | **Women**  **std** | **Men**  **mean** | **Men**  **std** |
| --- | --- | --- | --- | --- | --- | --- |
| **ctx-lh-bankssts** | 1.486 | 0.122 | 1.508 | 0.098 | 1.47 | 0.14 |
| **ctx-lh-caudalanteriorcingulate** | 1.28 | 0.158 | 1.321 | 0.178 | 1.249 | 0.141 |
| **ctx-lh-caudalmiddlefrontal** | 1.544 | 0.164 | 1.654 | 0.138 | 1.462 | 0.135 |
| **ctx-lh-cuneus** | 1.657 | 0.164 | 1.697 | 0.136 | 1.626 | 0.182 |
| **ctx-lh-entorhinal** | 0.977 | 0.104 | 0.975 | 0.092 | 0.979 | 0.116 |
| **ctx-lh-fusiform** | 1.316 | 0.1 | 1.321 | 0.086 | 1.313 | 0.113 |
| **ctx-lh-inferiorparietal** | 1.408 | 0.134 | 1.462 | 0.116 | 1.367 | 0.136 |
| **ctx-lh-inferiortemporal** | 1.296 | 0.13 | 1.339 | 0.133 | 1.264 | 0.124 |
| **ctx-lh-isthmuscingulate** | 1.506 | 0.17 | 1.546 | 0.164 | 1.477 | 0.175 |
| **ctx-lh-lateraloccipital** | 1.425 | 0.15 | 1.464 | 0.139 | 1.395 | 0.156 |
| **ctx-lh-lateralorbitofrontal** | 1.373 | 0.105 | 1.402 | 0.078 | 1.352 | 0.12 |
| **ctx-lh-lingual** | 1.596 | 0.134 | 1.615 | 0.136 | 1.582 | 0.137 |
| **ctx-lh-medialorbitofrontal** | 1.36 | 0.107 | 1.399 | 0.092 | 1.331 | 0.112 |
| **ctx-lh-middletemporal** | 1.292 | 0.145 | 1.362 | 0.139 | 1.24 | 0.132 |
| **ctx-lh-parahippocampal** | 1.107 | 0.098 | 1.103 | 0.095 | 1.111 | 0.105 |
| **ctx-lh-paracentral** | 1.452 | 0.13 | 1.515 | 0.139 | 1.404 | 0.105 |
| **ctx-lh-parsopercularis** | 1.491 | 0.129 | 1.541 | 0.122 | 1.454 | 0.126 |
| **ctx-lh-parsorbitalis** | 1.358 | 0.135 | 1.417 | 0.12 | 1.314 | 0.132 |
| **ctx-lh-parstriangularis** | 1.444 | 0.149 | 1.511 | 0.117 | 1.394 | 0.155 |
| **ctx-lh-pericalcarine** | 1.786 | 0.169 | 1.822 | 0.207 | 1.759 | 0.137 |
| **ctx-lh-postcentral** | 1.362 | 0.113 | 1.408 | 0.1 | 1.327 | 0.113 |
| **ctx-lh-posteriorcingulate** | 1.525 | 0.149 | 1.567 | 0.15 | 1.494 | 0.147 |
| **ctx-lh-precentral** | 1.44 | 0.12 | 1.499 | 0.096 | 1.395 | 0.121 |
| **ctx-lh-precuneus** | 1.632 | 0.154 | 1.683 | 0.145 | 1.593 | 0.156 |
| **ctx-lh-rostralanteriorcingulate** | 1.286 | 0.122 | 1.318 | 0.123 | 1.261 | 0.12 |
| **ctx-lh-rostralmiddlefrontal** | 1.483 | 0.165 | 1.581 | 0.136 | 1.41 | 0.149 |
| **ctx-lh-superiorfrontal** | 1.407 | 0.157 | 1.497 | 0.165 | 1.34 | 0.115 |
| **ctx-lh-superiorparietal** | 1.35 | 0.118 | 1.402 | 0.117 | 1.311 | 0.107 |
| **ctx-lh-superiortemporal** | 1.221 | 0.103 | 1.257 | 0.121 | 1.195 | 0.083 |
| **ctx-lh-supramarginal** | 1.378 | 0.135 | 1.429 | 0.11 | 1.339 | 0.143 |
| **ctx-lh-frontalpole** | 1.295 | 0.175 | 1.409 | 0.179 | 1.21 | 0.12 |
| **ctx-lh-temporalpole** | 0.95 | 0.089 | 0.979 | 0.089 | 0.927 | 0.087 |
| **ctx-lh-transversetemporal** | 1.6 | 0.165 | 1.609 | 0.21 | 1.593 | 0.132 |
| **ctx-lh-insula** | 1.273 | 0.089 | 1.284 | 0.089 | 1.266 | 0.092 |
| **ctx-rh-bankssts** | 1.517 | 0.115 | 1.545 | 0.119 | 1.496 | 0.113 |
| **ctx-rh-caudalanteriorcingulate** | 1.317 | 0.141 | 1.356 | 0.137 | 1.287 | 0.143 |
| **ctx-rh-caudalmiddlefrontal** | 1.539 | 0.179 | 1.648 | 0.174 | 1.458 | 0.14 |
| **ctx-rh-cuneus** | 1.701 | 0.153 | 1.684 | 0.196 | 1.714 | 0.119 |
| **ctx-rh-entorhinal** | 1.008 | 0.096 | 0.996 | 0.11 | 1.017 | 0.088 |
| **ctx-rh-fusiform** | 1.332 | 0.099 | 1.341 | 0.097 | 1.326 | 0.104 |
| **ctx-rh-inferiorparietal** | 1.416 | 0.144 | 1.471 | 0.138 | 1.376 | 0.14 |
| **ctx-rh-inferiortemporal** | 1.3 | 0.121 | 1.339 | 0.119 | 1.27 | 0.119 |
| **ctx-rh-isthmuscingulate** | 1.529 | 0.141 | 1.552 | 0.154 | 1.512 | 0.135 |
| **ctx-rh-lateraloccipital** | 1.445 | 0.152 | 1.493 | 0.125 | 1.41 | 0.165 |
| **ctx-rh-lateralorbitofrontal** | 1.395 | 0.102 | 1.423 | 0.08 | 1.374 | 0.115 |
| **ctx-rh-lingual** | 1.594 | 0.131 | 1.616 | 0.156 | 1.578 | 0.114 |
| **ctx-rh-medialorbitofrontal** | 1.386 | 0.11 | 1.427 | 0.091 | 1.356 | 0.117 |
| **ctx-rh-middletemporal** | 1.316 | 0.153 | 1.396 | 0.148 | 1.257 | 0.132 |
| **ctx-rh-parahippocampal** | 1.135 | 0.088 | 1.128 | 0.111 | 1.14 | 0.07 |
| **ctx-rh-paracentral** | 1.442 | 0.141 | 1.522 | 0.146 | 1.383 | 0.106 |
| **ctx-rh-parsopercularis** | 1.541 | 0.132 | 1.593 | 0.123 | 1.501 | 0.13 |
| **ctx-rh-parsorbitalis** | 1.384 | 0.155 | 1.458 | 0.127 | 1.328 | 0.155 |
| **ctx-rh-parstriangularis** | 1.467 | 0.16 | 1.549 | 0.144 | 1.406 | 0.149 |
| **ctx-rh-pericalcarine** | 1.832 | 0.171 | 1.877 | 0.184 | 1.799 | 0.16 |
| **ctx-rh-postcentral** | 1.368 | 0.12 | 1.429 | 0.089 | 1.322 | 0.124 |
| **ctx-rh-posteriorcingulate** | 1.541 | 0.153 | 1.599 | 0.17 | 1.497 | 0.128 |
| **ctx-rh-precentral** | 1.465 | 0.133 | 1.539 | 0.112 | 1.41 | 0.124 |
| **ctx-rh-precuneus** | 1.637 | 0.144 | 1.681 | 0.153 | 1.604 | 0.134 |
| **ctx-rh-rostralanteriorcingulate** | 1.285 | 0.113 | 1.345 | 0.121 | 1.24 | 0.087 |
| **ctx-rh-rostralmiddlefrontal** | 1.509 | 0.178 | 1.629 | 0.156 | 1.419 | 0.138 |
| **ctx-rh-superiorfrontal** | 1.418 | 0.158 | 1.515 | 0.161 | 1.346 | 0.115 |
| **ctx-rh-superiorparietal** | 1.347 | 0.119 | 1.402 | 0.12 | 1.306 | 0.104 |
| **ctx-rh-superiortemporal** | 1.26 | 0.105 | 1.298 | 0.122 | 1.232 | 0.086 |
| **ctx-rh-supramarginal** | 1.387 | 0.146 | 1.432 | 0.144 | 1.354 | 0.145 |
| **ctx-rh-frontalpole** | 1.352 | 0.162 | 1.462 | 0.153 | 1.27 | 0.117 |
| **ctx-rh-temporalpole** | 0.956 | 0.084 | 0.975 | 0.114 | 0.942 | 0.053 |
| **ctx-rh-transversetemporal** | 1.602 | 0.173 | 1.608 | 0.227 | 1.597 | 0.129 |
| **ctx-rh-insula** | 1.287 | 0.085 | 1.284 | 0.087 | 1.289 | 0.088 |

### CN to AD

## **Supplementary Table 18.** Mean and standard deviation of FDG SUVR within the “CN to AD” group.

|  | **All**  **mean** | **All**  **std** | **Women**  **mean** | **Women**  **std** | **Men**  **mean** | **Men**  **std** |
| --- | --- | --- | --- | --- | --- | --- |
| **ctx-lh-bankssts** | 1.519 | 0.148 | 1.544 | 0.176 | 1.489 | 0.113 |
| **ctx-lh-caudalanteriorcingulate** | 1.304 | 0.14 | 1.323 | 0.11 | 1.282 | 0.175 |
| **ctx-lh-caudalmiddlefrontal** | 1.562 | 0.181 | 1.591 | 0.198 | 1.53 | 0.167 |
| **ctx-lh-cuneus** | 1.678 | 0.19 | 1.674 | 0.159 | 1.683 | 0.234 |
| **ctx-lh-entorhinal** | 1.003 | 0.089 | 1.005 | 0.097 | 1.002 | 0.085 |
| **ctx-lh-fusiform** | 1.354 | 0.104 | 1.385 | 0.083 | 1.318 | 0.12 |
| **ctx-lh-inferiorparietal** | 1.444 | 0.144 | 1.488 | 0.151 | 1.393 | 0.126 |
| **ctx-lh-inferiortemporal** | 1.318 | 0.113 | 1.331 | 0.084 | 1.304 | 0.145 |
| **ctx-lh-isthmuscingulate** | 1.536 | 0.144 | 1.55 | 0.159 | 1.519 | 0.135 |
| **ctx-lh-lateraloccipital** | 1.475 | 0.139 | 1.484 | 0.093 | 1.463 | 0.186 |
| **ctx-lh-lateralorbitofrontal** | 1.404 | 0.104 | 1.407 | 0.096 | 1.401 | 0.12 |
| **ctx-lh-lingual** | 1.608 | 0.121 | 1.606 | 0.121 | 1.61 | 0.13 |
| **ctx-lh-medialorbitofrontal** | 1.368 | 0.116 | 1.385 | 0.109 | 1.348 | 0.129 |
| **ctx-lh-middletemporal** | 1.322 | 0.14 | 1.357 | 0.092 | 1.282 | 0.18 |
| **ctx-lh-parahippocampal** | 1.13 | 0.089 | 1.141 | 0.08 | 1.117 | 0.103 |
| **ctx-lh-paracentral** | 1.494 | 0.151 | 1.537 | 0.18 | 1.445 | 0.102 |
| **ctx-lh-parsopercularis** | 1.521 | 0.172 | 1.549 | 0.183 | 1.49 | 0.166 |
| **ctx-lh-parsorbitalis** | 1.39 | 0.154 | 1.38 | 0.156 | 1.402 | 0.164 |
| **ctx-lh-parstriangularis** | 1.466 | 0.181 | 1.494 | 0.221 | 1.434 | 0.132 |
| **ctx-lh-pericalcarine** | 1.772 | 0.147 | 1.728 | 0.162 | 1.822 | 0.119 |
| **ctx-lh-postcentral** | 1.399 | 0.152 | 1.422 | 0.175 | 1.373 | 0.131 |
| **ctx-lh-posteriorcingulate** | 1.551 | 0.156 | 1.586 | 0.17 | 1.511 | 0.139 |
| **ctx-lh-precentral** | 1.491 | 0.17 | 1.526 | 0.189 | 1.452 | 0.15 |
| **ctx-lh-precuneus** | 1.663 | 0.147 | 1.702 | 0.169 | 1.617 | 0.112 |
| **ctx-lh-rostralanteriorcingulate** | 1.3 | 0.101 | 1.306 | 0.097 | 1.293 | 0.113 |
| **ctx-lh-rostralmiddlefrontal** | 1.512 | 0.145 | 1.55 | 0.141 | 1.468 | 0.148 |
| **ctx-lh-superiorfrontal** | 1.426 | 0.137 | 1.461 | 0.132 | 1.386 | 0.142 |
| **ctx-lh-superiorparietal** | 1.391 | 0.161 | 1.433 | 0.167 | 1.342 | 0.152 |
| **ctx-lh-superiortemporal** | 1.271 | 0.14 | 1.289 | 0.109 | 1.25 | 0.176 |
| **ctx-lh-supramarginal** | 1.408 | 0.158 | 1.449 | 0.175 | 1.362 | 0.132 |
| **ctx-lh-frontalpole** | 1.334 | 0.16 | 1.365 | 0.157 | 1.298 | 0.167 |
| **ctx-lh-temporalpole** | 0.965 | 0.09 | 0.983 | 0.094 | 0.945 | 0.089 |
| **ctx-lh-transversetemporal** | 1.672 | 0.214 | 1.707 | 0.211 | 1.632 | 0.228 |
| **ctx-lh-insula** | 1.288 | 0.113 | 1.307 | 0.099 | 1.267 | 0.132 |
| **ctx-rh-bankssts** | 1.526 | 0.144 | 1.511 | 0.139 | 1.542 | 0.158 |
| **ctx-rh-caudalanteriorcingulate** | 1.3 | 0.137 | 1.333 | 0.132 | 1.263 | 0.144 |
| **ctx-rh-caudalmiddlefrontal** | 1.553 | 0.159 | 1.564 | 0.159 | 1.54 | 0.171 |
| **ctx-rh-cuneus** | 1.695 | 0.142 | 1.669 | 0.112 | 1.725 | 0.174 |
| **ctx-rh-entorhinal** | 0.943 | 0.095 | 0.896 | 0.093 | 0.997 | 0.069 |
| **ctx-rh-fusiform** | 1.322 | 0.108 | 1.313 | 0.114 | 1.333 | 0.109 |
| **ctx-rh-inferiorparietal** | 1.431 | 0.156 | 1.44 | 0.163 | 1.42 | 0.159 |
| **ctx-rh-inferiortemporal** | 1.276 | 0.122 | 1.252 | 0.109 | 1.304 | 0.138 |
| **ctx-rh-isthmuscingulate** | 1.558 | 0.134 | 1.566 | 0.157 | 1.549 | 0.114 |
| **ctx-rh-lateraloccipital** | 1.461 | 0.161 | 1.448 | 0.138 | 1.476 | 0.195 |
| **ctx-rh-lateralorbitofrontal** | 1.396 | 0.105 | 1.367 | 0.093 | 1.429 | 0.115 |
| **ctx-rh-lingual** | 1.61 | 0.1 | 1.587 | 0.097 | 1.636 | 0.105 |
| **ctx-rh-medialorbitofrontal** | 1.389 | 0.103 | 1.402 | 0.086 | 1.373 | 0.124 |
| **ctx-rh-middletemporal** | 1.313 | 0.145 | 1.311 | 0.108 | 1.315 | 0.188 |
| **ctx-rh-parahippocampal** | 1.107 | 0.076 | 1.084 | 0.077 | 1.132 | 0.072 |
| **ctx-rh-paracentral** | 1.491 | 0.157 | 1.537 | 0.176 | 1.438 | 0.125 |
| **ctx-rh-parsopercularis** | 1.535 | 0.17 | 1.502 | 0.168 | 1.573 | 0.177 |
| **ctx-rh-parsorbitalis** | 1.437 | 0.211 | 1.409 | 0.225 | 1.469 | 0.207 |
| **ctx-rh-parstriangularis** | 1.479 | 0.181 | 1.458 | 0.175 | 1.503 | 0.199 |
| **ctx-rh-pericalcarine** | 1.812 | 0.15 | 1.757 | 0.144 | 1.876 | 0.139 |
| **ctx-rh-postcentral** | 1.401 | 0.145 | 1.428 | 0.158 | 1.37 | 0.133 |
| **ctx-rh-posteriorcingulate** | 1.531 | 0.176 | 1.574 | 0.199 | 1.482 | 0.145 |
| **ctx-rh-precentral** | 1.475 | 0.154 | 1.492 | 0.161 | 1.455 | 0.156 |
| **ctx-rh-precuneus** | 1.652 | 0.152 | 1.687 | 0.181 | 1.611 | 0.111 |
| **ctx-rh-rostralanteriorcingulate** | 1.282 | 0.118 | 1.31 | 0.136 | 1.25 | 0.092 |
| **ctx-rh-rostralmiddlefrontal** | 1.522 | 0.142 | 1.537 | 0.13 | 1.506 | 0.164 |
| **ctx-rh-superiorfrontal** | 1.424 | 0.126 | 1.448 | 0.129 | 1.397 | 0.128 |
| **ctx-rh-superiorparietal** | 1.362 | 0.138 | 1.388 | 0.147 | 1.332 | 0.131 |
| **ctx-rh-superiortemporal** | 1.273 | 0.142 | 1.258 | 0.115 | 1.291 | 0.177 |
| **ctx-rh-supramarginal** | 1.388 | 0.153 | 1.402 | 0.156 | 1.371 | 0.161 |
| **ctx-rh-frontalpole** | 1.332 | 0.158 | 1.327 | 0.166 | 1.338 | 0.162 |
| **ctx-rh-temporalpole** | 0.913 | 0.112 | 0.88 | 0.108 | 0.95 | 0.113 |
| **ctx-rh-transversetemporal** | 1.614 | 0.212 | 1.601 | 0.209 | 1.628 | 0.232 |
| **ctx-rh-insula** | 1.277 | 0.112 | 1.258 | 0.092 | 1.298 | 0.135 |

### AD

## **Supplementary Table 19.** Mean and standard deviation of FDG SUVR within the “AD” group.

|  | **All**  **mean** | **All**  **std** | **Women**  **mean** | **Women**  **std** | **Men**  **mean** | **Men**  **std** |
| --- | --- | --- | --- | --- | --- | --- |
| **ctx-lh-bankssts** | 1.367 | 0.209 | 1.342 | 0.216 | 1.391 | 0.2 |
| **ctx-lh-caudalanteriorcingulate** | 1.307 | 0.157 | 1.323 | 0.122 | 1.293 | 0.183 |
| **ctx-lh-caudalmiddlefrontal** | 1.448 | 0.204 | 1.422 | 0.199 | 1.472 | 0.207 |
| **ctx-lh-cuneus** | 1.616 | 0.234 | 1.616 | 0.231 | 1.617 | 0.239 |
| **ctx-lh-entorhinal** | 0.914 | 0.116 | 0.902 | 0.108 | 0.926 | 0.123 |
| **ctx-lh-fusiform** | 1.271 | 0.138 | 1.268 | 0.149 | 1.273 | 0.127 |
| **ctx-lh-inferiorparietal** | 1.239 | 0.21 | 1.229 | 0.212 | 1.249 | 0.209 |
| **ctx-lh-inferiortemporal** | 1.168 | 0.155 | 1.154 | 0.16 | 1.181 | 0.151 |
| **ctx-lh-isthmuscingulate** | 1.344 | 0.169 | 1.327 | 0.177 | 1.36 | 0.161 |
| **ctx-lh-lateraloccipital** | 1.353 | 0.208 | 1.349 | 0.225 | 1.357 | 0.193 |
| **ctx-lh-lateralorbitofrontal** | 1.384 | 0.156 | 1.362 | 0.137 | 1.405 | 0.17 |
| **ctx-lh-lingual** | 1.572 | 0.178 | 1.59 | 0.19 | 1.555 | 0.165 |
| **ctx-lh-medialorbitofrontal** | 1.355 | 0.144 | 1.344 | 0.118 | 1.364 | 0.164 |
| **ctx-lh-middletemporal** | 1.172 | 0.165 | 1.162 | 0.174 | 1.182 | 0.157 |
| **ctx-lh-parahippocampal** | 1.072 | 0.1 | 1.085 | 0.092 | 1.061 | 0.107 |
| **ctx-lh-paracentral** | 1.477 | 0.159 | 1.476 | 0.151 | 1.478 | 0.168 |
| **ctx-lh-parsopercularis** | 1.472 | 0.183 | 1.458 | 0.167 | 1.485 | 0.197 |
| **ctx-lh-parsorbitalis** | 1.385 | 0.202 | 1.381 | 0.188 | 1.389 | 0.216 |
| **ctx-lh-parstriangularis** | 1.435 | 0.195 | 1.43 | 0.175 | 1.439 | 0.212 |
| **ctx-lh-pericalcarine** | 1.772 | 0.245 | 1.776 | 0.249 | 1.768 | 0.242 |
| **ctx-lh-postcentral** | 1.355 | 0.154 | 1.35 | 0.147 | 1.359 | 0.161 |
| **ctx-lh-posteriorcingulate** | 1.456 | 0.156 | 1.457 | 0.141 | 1.455 | 0.169 |
| **ctx-lh-precentral** | 1.451 | 0.161 | 1.44 | 0.159 | 1.461 | 0.164 |
| **ctx-lh-precuneus** | 1.494 | 0.202 | 1.476 | 0.189 | 1.51 | 0.214 |
| **ctx-lh-rostralanteriorcingulate** | 1.306 | 0.156 | 1.305 | 0.148 | 1.307 | 0.163 |
| **ctx-lh-rostralmiddlefrontal** | 1.437 | 0.196 | 1.432 | 0.181 | 1.442 | 0.21 |
| **ctx-lh-superiorfrontal** | 1.392 | 0.153 | 1.384 | 0.137 | 1.4 | 0.167 |
| **ctx-lh-superiorparietal** | 1.277 | 0.178 | 1.263 | 0.177 | 1.29 | 0.179 |
| **ctx-lh-superiortemporal** | 1.206 | 0.137 | 1.189 | 0.134 | 1.221 | 0.139 |
| **ctx-lh-supramarginal** | 1.277 | 0.182 | 1.269 | 0.176 | 1.284 | 0.187 |
| **ctx-lh-frontalpole** | 1.287 | 0.196 | 1.289 | 0.178 | 1.284 | 0.212 |
| **ctx-lh-temporalpole** | 0.941 | 0.104 | 0.94 | 0.1 | 0.943 | 0.109 |
| **ctx-lh-transversetemporal** | 1.64 | 0.227 | 1.631 | 0.209 | 1.648 | 0.244 |
| **ctx-lh-insula** | 1.282 | 0.128 | 1.27 | 0.108 | 1.292 | 0.143 |
| **ctx-rh-bankssts** | 1.416 | 0.193 | 1.393 | 0.184 | 1.437 | 0.2 |
| **ctx-rh-caudalanteriorcingulate** | 1.336 | 0.147 | 1.345 | 0.129 | 1.328 | 0.162 |
| **ctx-rh-caudalmiddlefrontal** | 1.462 | 0.202 | 1.447 | 0.2 | 1.475 | 0.205 |
| **ctx-rh-cuneus** | 1.631 | 0.228 | 1.622 | 0.248 | 1.64 | 0.21 |
| **ctx-rh-entorhinal** | 0.921 | 0.113 | 0.907 | 0.106 | 0.933 | 0.118 |
| **ctx-rh-fusiform** | 1.287 | 0.128 | 1.28 | 0.136 | 1.294 | 0.12 |
| **ctx-rh-inferiorparietal** | 1.27 | 0.216 | 1.25 | 0.213 | 1.289 | 0.219 |
| **ctx-rh-inferiortemporal** | 1.195 | 0.146 | 1.174 | 0.151 | 1.214 | 0.14 |
| **ctx-rh-isthmuscingulate** | 1.383 | 0.178 | 1.359 | 0.171 | 1.406 | 0.183 |
| **ctx-rh-lateraloccipital** | 1.377 | 0.208 | 1.365 | 0.225 | 1.388 | 0.193 |
| **ctx-rh-lateralorbitofrontal** | 1.393 | 0.146 | 1.369 | 0.134 | 1.415 | 0.154 |
| **ctx-rh-lingual** | 1.583 | 0.178 | 1.592 | 0.186 | 1.575 | 0.17 |
| **ctx-rh-medialorbitofrontal** | 1.381 | 0.148 | 1.368 | 0.129 | 1.392 | 0.164 |
| **ctx-rh-middletemporal** | 1.214 | 0.163 | 1.197 | 0.16 | 1.23 | 0.166 |
| **ctx-rh-parahippocampal** | 1.101 | 0.097 | 1.115 | 0.087 | 1.089 | 0.103 |
| **ctx-rh-paracentral** | 1.478 | 0.157 | 1.473 | 0.147 | 1.484 | 0.167 |
| **ctx-rh-parsopercularis** | 1.515 | 0.182 | 1.504 | 0.17 | 1.525 | 0.193 |
| **ctx-rh-parsorbitalis** | 1.42 | 0.199 | 1.407 | 0.199 | 1.432 | 0.2 |
| **ctx-rh-parstriangularis** | 1.461 | 0.188 | 1.454 | 0.171 | 1.468 | 0.203 |
| **ctx-rh-pericalcarine** | 1.819 | 0.254 | 1.823 | 0.256 | 1.815 | 0.255 |
| **ctx-rh-postcentral** | 1.375 | 0.162 | 1.367 | 0.156 | 1.382 | 0.168 |
| **ctx-rh-posteriorcingulate** | 1.474 | 0.164 | 1.474 | 0.15 | 1.473 | 0.178 |
| **ctx-rh-precentral** | 1.465 | 0.16 | 1.455 | 0.162 | 1.475 | 0.158 |
| **ctx-rh-precuneus** | 1.505 | 0.205 | 1.486 | 0.191 | 1.523 | 0.216 |
| **ctx-rh-rostralanteriorcingulate** | 1.324 | 0.156 | 1.327 | 0.156 | 1.32 | 0.156 |
| **ctx-rh-rostralmiddlefrontal** | 1.458 | 0.201 | 1.45 | 0.19 | 1.465 | 0.212 |
| **ctx-rh-superiorfrontal** | 1.406 | 0.158 | 1.402 | 0.147 | 1.409 | 0.168 |
| **ctx-rh-superiorparietal** | 1.281 | 0.195 | 1.261 | 0.197 | 1.299 | 0.192 |
| **ctx-rh-superiortemporal** | 1.242 | 0.135 | 1.223 | 0.129 | 1.259 | 0.139 |
| **ctx-rh-supramarginal** | 1.313 | 0.182 | 1.301 | 0.172 | 1.325 | 0.191 |
| **ctx-rh-frontalpole** | 1.322 | 0.195 | 1.324 | 0.181 | 1.32 | 0.207 |
| **ctx-rh-temporalpole** | 0.941 | 0.102 | 0.934 | 0.104 | 0.947 | 0.101 |
| **ctx-rh-transversetemporal** | 1.63 | 0.204 | 1.626 | 0.182 | 1.633 | 0.223 |
| **ctx-rh-insula** | 1.293 | 0.123 | 1.282 | 0.109 | 1.304 | 0.134 |
